# Aurantio-obtusin improves obesity and protects hepatic inflammation by rescuing mitochondrial damage in overwhelmed brown adipose tissue

**DOI:** 10.1186/s13020-025-01097-y

**Published:** 2025-03-25

**Authors:** Ruiyu Wu, Runping Liu, Ranyun Chen, Yijie Li, Xiaoyong Xue, Yinhao Zhang, Fanghong Li, Jiaorong Qu, Lingling Qin, Chen Wang, Xiaojiaoyang Li

**Affiliations:** 1 School of Chinese Materia Medica, Beijing University of Chinese Medicine, 11 Bei San Huan Dong Lu, Beijing, 100029 China; 2 School of Life Sciences, Beijing University of Chinese Medicine, 11 Bei San Huan Dong Lu, Beijing, 100029 China; 3 Department of Science and Technology, Beijing University of Chinese Medicine, 11 Bei San Huan Dong Lu, Beijing, 100029 China

**Keywords:** Obesity, Aurantio-obtusin, Brown adipose tissue, Mitochondrial DNA, Inflammation

## Abstract

**Background:**

Obesity is frequently linked to chronic systamic inflammation and presents significant challenges to public health. Aurantio-obtusin (AO) boosted the brown adipose tissue (BAT) thermogenesis in diet-induced obesity. However, the specific mechanisms by which injured mitochondria-related damage signals derived from overwhelmed BAT can transmit to liver and exacerbate metabolic disorders and whether AO can reverse this process remain unknown.

**Materials and methods:**

After applying high-fat diet and glucose-fructose water (HFHS)-induced obesity mice, different BAT transplant procedures and primary BAT adipocytes, we investigated the anti-obesity effects and mechanism of AO through RNA sequencing and biology techniques.

**Results:**

AO improved whole-body lipid accumulation, mitochondrial metabolism in BAT and hepatic inflammation in HFHS-induced obesity mice. Interscapular transplant of BAT-derived from obese donor mice triggered hepatic inflammation of chow diet-fed recipient mice, which was protected by AO. Furthermore, the transplantation of BAT-derived from AO-treated mice protected hepatic inflammation in obese mice. In vivo and in lipid-challenged primary BAT adipocytes, AO decreased kexin type 9 (PCSK9), prevented mPTP opening and mitochondrial DNA (mtDNA) release in extracellular vesicles (EVs) manner by inhibiting the acetylation of cyclophilin D associated with adenine nucleotide translocase, suppressing oligomerization of voltage-dependent anion channel 1 and activating mitophagy. Ultimately, AO inhibited mtDNA-containing EVs-induced cyclic GMP-AMP synthase/stimulator of interferon genes (STING) activation and hepatic inflammation, which was confirmed by Sting^−/−^ mice.

**Conclusion:**

AO not only improves thermogenesis and mitochondrial function of BAT but also prevents liver inflammation by repairing mitochondrial function and blocking the transfer of mtDNA from BAT to the liver.

**Graphic abstract:**

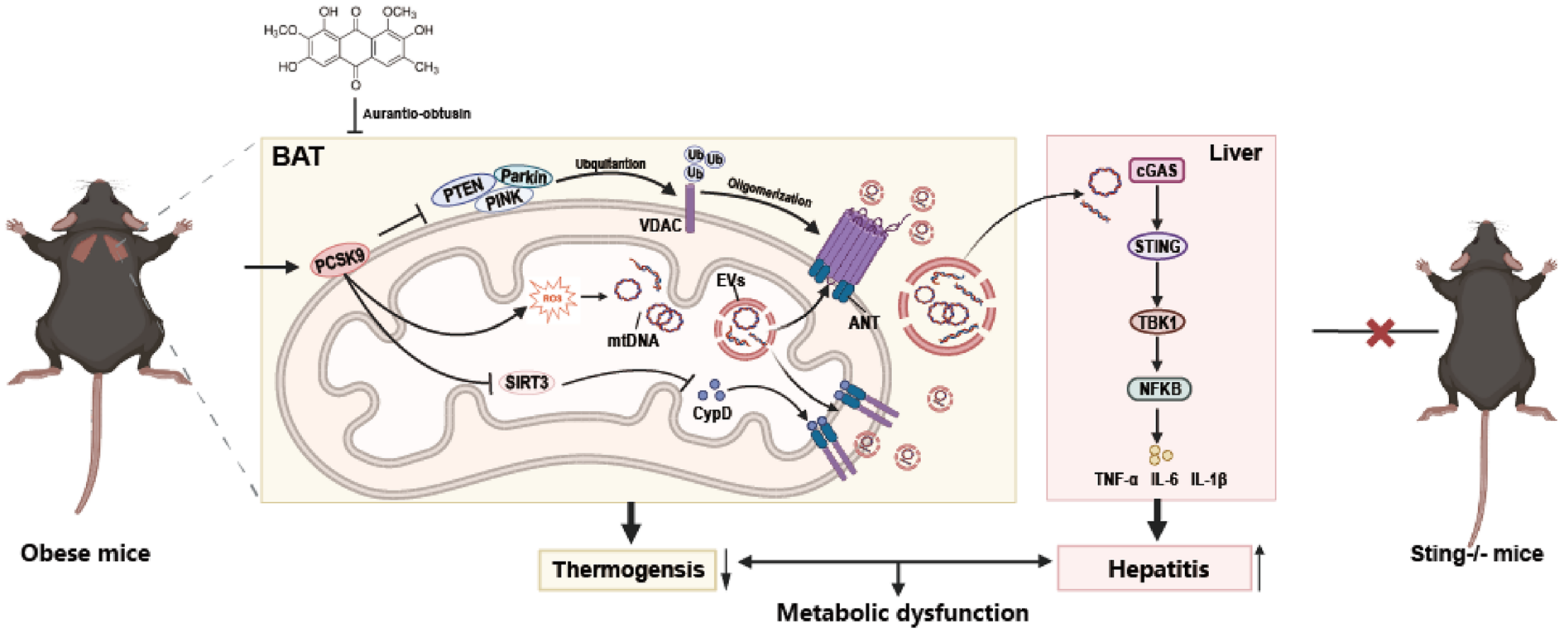

**Supplementary Information:**

The online version contains supplementary material available at 10.1186/s13020-025-01097-y.

## Introduction

As recognized as a global epidemic by the World Health Organization, obesity is characterized by an imbalance between food intake and the body's energy expenditure, regularly due to abnormal metabolism and frequently associated with chronic, low-grade systemic inflammation. This imbalance leads to the excessive accumulation of fat in the whole body, resulting in uncontrolled weight gain and a range of associated metabolic diseases including but not limited to cardiovascular diseases, metabolic dysfunction associated with fatty liver disease, insulin resistance and diabetes [1]. Alarmingly, it is predicted that 57.8% of the world's adult population will be overweight or obese by 2030, which will become a huge threat to human wellness and an enormous financial burden [2]. With ongoing advancements in obesity research, it has been found that obesity is linked to worse outcomes in various chronic inflammatory disorders and is becoming more widely acknowledged as an abnormal state of excessive accumulation or abnormal distribution of body fat [3]. Adipose tissue is an organ with multiple functions that regulate metabolic homeostasis throughout the body and generates a variety of adipokines, cytokines, and other endocrine chemicals. Thus, a comprehensive understanding of the intricate relationships that exist between adipose tissues and other metabolic organs is essential to comprehending energy balance and the emergence of obesity.

Recent studies indicate that molecules released by brown adipose tissue (BAT) play a crucial function in regulating the metabolic phenotype of other critical organs such as white adipose tissue (WAT). In response to thermogenic stimulation, fibroblast growth factor 21 and C-X-C motif chemokine ligand-14 were released from BAT and caused the browning process of WAT in mice [4, 5]. Unlike WAT, the liver plays a critical role in lipogenesis, gluconeogenesis, and cholesterol metabolism. As time progressed, the literature has increasingly focused on the complex inter-regulatory relationship mechanism between BAT and the liver. Scheja L et al*.* revealed that BAT cells possess the ability to modulate hepatic metabolism through the synthesis and secretion of insulin-like growth factor 1, which leads to a reduction in plasma glucagon levels [6]. Additionally, another study reported that neuregulin 4, a hormonal checkpoint secreted during BAT cell differentiation, reduced fat synthesis signaling in the liver, thereby preventing unhealthy diet-induced insulin resistance and hepatic steatosis [7]. Our recent study also demonstrated that the inhibition of peroxisome proliferator-activated receptor α (Ppara) signaling pathway and lipid metabolic abnormalities in liver were observed in mice fed high-fat diet and glucose-fructose water (HFHS) following BAT removal [8]. Besides, mitochondria are essential for adipocyte activity since they are the organelles responsible for critical metabolic pathways such as β-oxidation and ATP production [9]. From the perspective of damaging mitochondria, abnormal BAT damage and hepatic lipid deposition may share a mechanistic intersection since excessive reactive oxygen species (ROS) production and accumulation in BAT cells can promote mitochondrial oxidative damage while higher free fatty acid or other lipids in hepatocytes can lead to lipotoxicity and aggravate inflammation [10]. However, whether and how mitochondria in BAT undergo morphological changes and release damaging substances to aggravate liver injury is presently uncertain in the field of obesity.

Aurantio-obtusin (AO), a peculiar anthraquinone component extracted from cassia seeds, has been recognized for its diverse pharmacological effects, including anti-inflammatory, antioxidant and antihypertensive properties [11]. Notably, in recent years, a variety of studies examining the effects of AO on abnormal lipid metabolism and obesity-associated diseases have attracted considerable research attention [12]. Reports indicate that AO can improve lipid accumulation in WAT, reduce both the volume and weight of WAT, and even encourage WAT browning in mice [8]. Additionally, AO has been shown to activate lipid metabolism and thermogenesis in BAT and improve liver function by activating autophagy and reducing lipid levels in the livers of mice with obesity-associated hepatitis [13, 14]. Furthermore, we recently discovered that AO could enhance the enzymatic activities of mitochondrial complexes I and IV in BATs. Notably, after the removal of interscapular BAT, AO was unable to reduce lipid accumulation or improve metabolic abnormalities in the livers of obese mice [8]. However, the mechanisms by which AO regulates the pathophysiological communication between BAT and liver, as well as the potential effects on mitochondrial dysfunction in BAT remain largely unexplored.

In this study, we investigated the critical role of BAT in the anti-obesity and liver inflammation-mitigating effects of AO by employing an HFHS-fed mouse obese model and a BAT excision-transplantation model. Additionally, we confirmed the protective effects of AO against mitochondrial damage in overwhelmed BAT using primary BAT cells, and further elucidated the specific mechanism by which AO inhibited the transmission of damage signals through EVs to activate /stimulator of interferon genes (STING)-dependent inflammation in the liver. Our study provides new insights into the mechanisms of inter-tissue damage communication in obesity and further elucidates the mechanisms by which AO protects against obesity from this perspective.

## Methods and materials

### Materials

AO (PS0100) was acquired from Push Bio-Technology (Chengdu, China). Oleic acid (C4977) was obtained from APExBIO (Houston, TX, USA) and palmitic acid (P101061) was purchased from Aladdin (Shanghai, China). Glucose (B21882), fructose (B21896), GW4869 (S81516) and RNase A (BN20090) were obtained from Yuanye Bio-Technology (Shanghai, China). DNase (10,104,159,001) was purchased from Roche.

### Animal studies

**Fig. 1:**
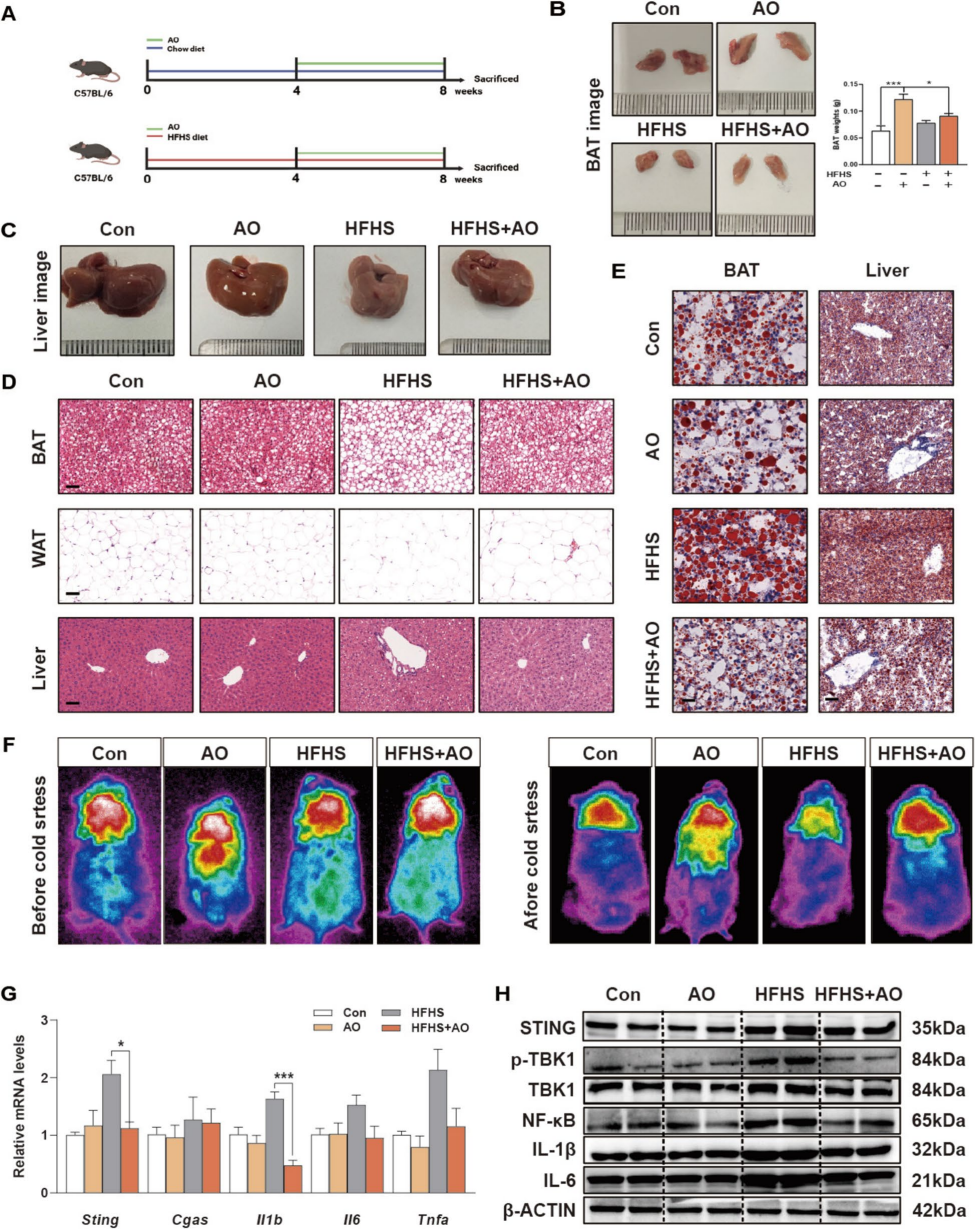
AO improves BAT thermogenesis and liver inflammation in HFHS diet-fed mice. **A** Mice were fed a chow or HFHS diet for 4 weeks before receiving AO (10 mg/kg) or vehicle by oral gavage for 4 weeks. **B** Representative images of BAT and BAT weights. **C** Representative images of liver. **D** H&E images of BAT, WAT, and liver tissues. Scale bar = 40 μm (BAT and WAT) or 20 μm (Liver). **E** Oil Red O staining of BAT and liver. Scale bar = 20 μm. **F** The mice of infrared thermography before and after acute freezing stress test. **G** The mRNA level of *Sting, Cgas, Il1b, Il6* and *Tnfα* were determined by PCR and normalized with *Hprt1* in livers. **H** Protein levels of STING, P-TBK1, TBK1, NF-ĸB, IL-1β and IL-6 were tested in livers by western blot analysis and normalized with β-ACTIN. Statistical significance: *P < 0.05, ***P < 0.001, compared between groups (n = 6)

Male C57BL/6 J mice (18–22 g, 5–7 weeks old) were purchased from Vita River Laboratory Animal Technology (Beijing, China). All mice were acclimatized in a stable environment (22 ± 2 °C, 40 ± 10% humidity, 12-h light/12-h dark cycle) with free access to food and water ad libitum. All animal studies and procedures were complied with the guidelines of the Institutional Animal Care and Use Committee of Beijing University of Chinese Medicine. After seven days of adaptive feeding, mice were randomly divided into 4 groups (n = 6) in Fig. 1A: (1) control group (chow diet); (2) AO (10 mg/kg) with chow diet group; (3) HFHS model group; (4) HFHS diet with AO (10 mg/kg) treatment group. HFHS model was induced by a high-fat diet and high-fructose-glucose solution (D-glucose:18.9 g/L and D-fructose: 23.1 g/L, with 42% of calories from lipids and 0.2% cholesterol) prepared in drinking water [15]. Based on data from our previous research, we chose AO (10 mg/kg) as the safe and effective dosage for the entire study. Subsequently, after being fed with a chow diet or HFHS diet for 4 weeks, mice in groups (2) and (4) were simultaneously given AO once daily by oral administration for another 4 weeks. Then mice in groups (1) and (3) were orally treated with vehicle solution.

**Fig. 2:**
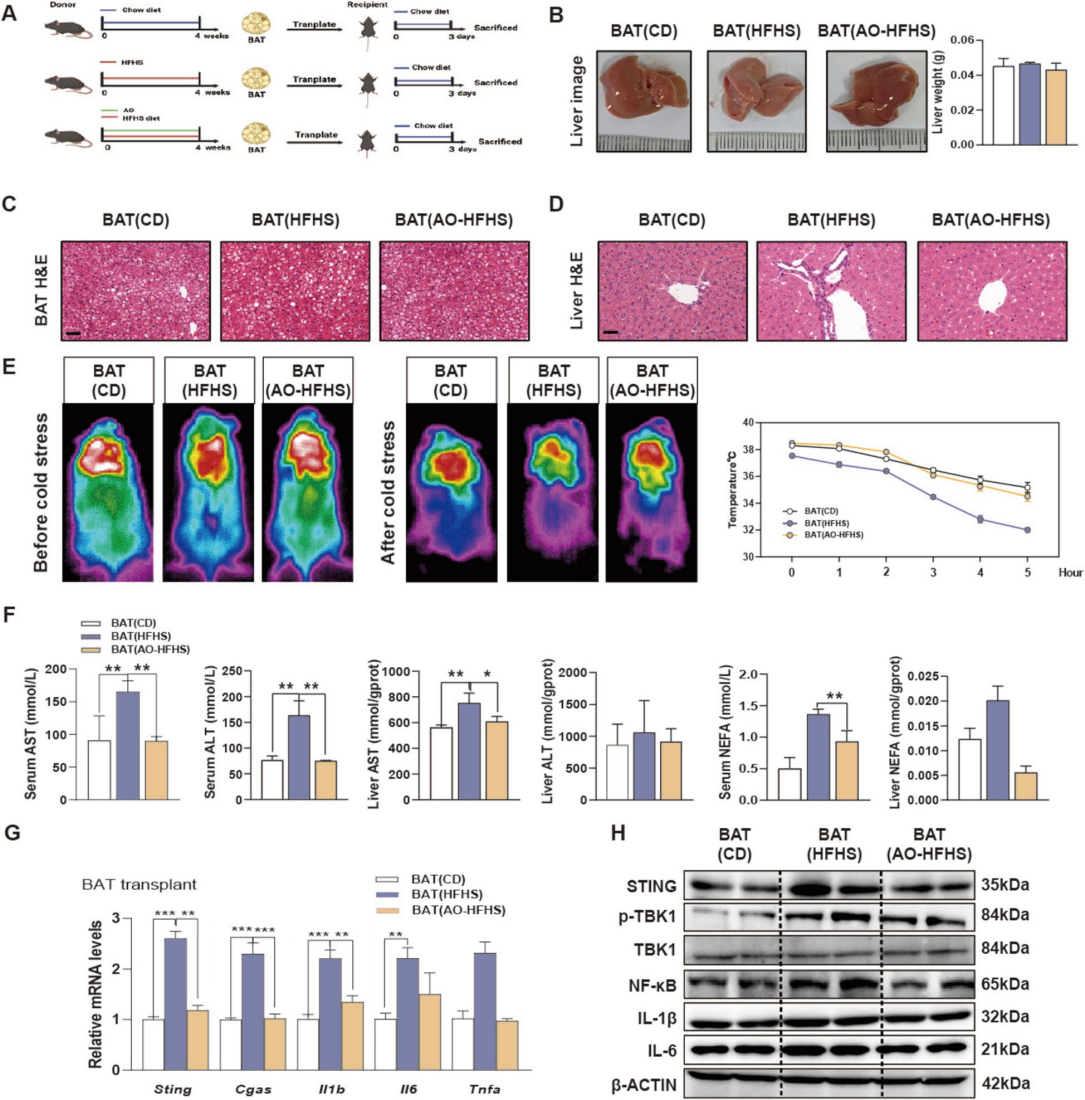
AO treatment in donor mice protects obese mice-derived BAT-induced hepatic inflammation in chow diet-fed recipient mice. **A** Mice were given with AO (10 mg/kg) or HFHS for 4 weeks for BAT collection, which were then transplanted into the control mice and sacrificed after 3 days. **B** Representative images and weights of livers. **C** H & E images of BAT. Scale bar = 40 μm. **D** H & E images of liver. Scale bar = 40 μm. **E** Representative infrared images of interscapular temperature and line chart of anal temperature after cold stress. **F** Serum and hepatic levels of AST, ALT and NEFA. **G** Relative mRNA levels of *Sting*, *Cgas*, *Il1β*, *Il6* and *Tnfα* were determined by qPCR and normalized using *Hprt1* as an internal control in livers in BAT transplant group. **H** Representative immunoblots against STING, P-TBK1, TBK1, NF-ĸB, IL-1β, IL-6 and β-ACTIN were shown in livers. Statistical significance: The abbreviations used in this figure refer to recipient mice that received transplants of BAT derived from control, obese or AO-treated obese mice, namely BAT (CD), BAT (HFHS) and BAT (AO-HFHS). *P < 0.05, **P < 0.01, ***P < 0.001, compared between groups (n = 6)

**Fig. 3:**
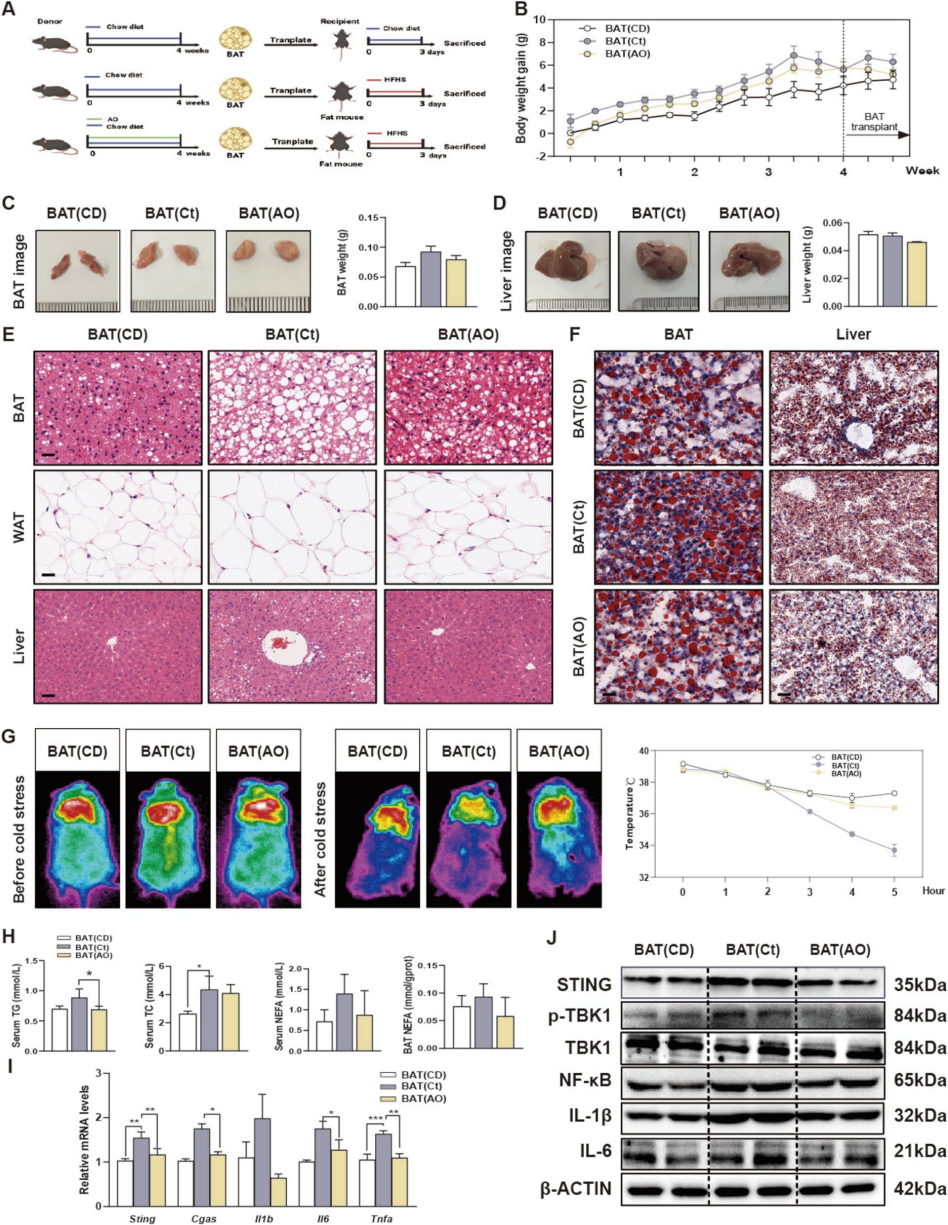
The transplantation of AO-stimulated BAT alleviates liver inflammation caused by lipid toxicity. **A** Mice were given with AO (10 mg/kg) and chow diet for 4 weeks for BAT collection, which were then transplanted into obese mice and sacrificed after 3 days. **B** Line chart of body weight change in mice. **C** Representative images of BAT and BAT weight. **D** Liver weight and images. **E** Representative images of H & E staining of BAT, WAT and liver. Scale bar = 40 μm in BAT and WAT, scale bar = 20 μm in liver. **F** Oil red O staining images of BAT and liver. Scale bar = 40 μm **G** Representative images and the changes of rectal temperature after freezing stress. **H** TG, TC, NEFA levels in serum and NEFA levels in BAT. **I** The mRNA levels of *Sting*, *Cgas*, *Il1β*, *Il6* and *Tnfa* in liver tissues were determined by qPCR and normalized using *Hprt1* as an internal control. **J** Representative immunoblots against STING, P-TBK1, TBK1, NF-ĸB, IL-1β, IL-6 and β-ACTIN were shown in livers. The abbreviations used in this figure refer to recipient mice were fed with an HFHS diet while simultaneously transplanting BAT from control or AO-treated chow diet-fed mice, namely BAT (CD), BAT (Ct) or BAT (AO). Statistical significance: *P < 0.05, *P < 0.05, **P < 0.01, ***P < 0.001, compared between groups (n = 6)

For BAT transplant surgeries, the BATs of donor mice were dissected after euthanization by isoflurane and placed in sterile warm saline. The BAT pieces were implanted subcutaneously on either side of the scapular region of the recipient mice through bilateral small incisions, each measuring 2–3 mm in length. The whole procedures were processed as quickly as possible under isoflurane anesthesia [16]. For WAT transplant surgeries, epididymal white adipose tissue (eWAT) was isolated from donor mice and maintained in sterile saline for less than 5 min prior to transplantation. Similarly, the donor WAT slices were carefully lodged deep between the folds of the endogenous epididymal fat in the recipient mice [17]. In the first batch of the transplantation experiment (Fig. 2A), donor mice were distributed into 3 groups (n = 6): (1) control group (chow diet); (2) HFHS model group; (3) HFHS diet with AO (10 mg/kg) treatment group. Mice in groups 2 and 3 were fed with HFHS diet or HFHS diet plus AO treatment for 4 weeks, then were removed BAT and transplanted into recipient mice with chow diet (An incision was made above the interscapular BAT in each recipient mouse scapula, which was then placed the BAT isolated from the donor mice). The recipient mice were sacrificed after 3 days. In the second and third batches of transplantation experiments (Fig. 3A and Fig. S4A), donor mice were randomized into 3 groups (n = 6): (1 and 2) control group (chow diet); (3) AO (10 mg/kg) with chow diet group. Meanwhile, recipient mice were also divided optionally into 3 groups: (1) control group (chow diet); (2 and 3) HFHS diet groups. After given AO treatment for 4 weeks, donor mice were removed BAT or WAT and transplanted BAT (transplantation method was the same as above) or WAT (Collected WAT from donor mice was placed in the inguinal white fat of the recipient mice) into recipient mice. Three days later, recipient mice were killed after the transplantation surgery. In the EV injection experiment (Fig. 5F), mice were distributed into 5 groups (n = 6): (1) EV-control group; (2) EV-oleic acid and palmitic acid (OAPA) group; (3) EV-OAPA + AO group; (4) EV-OAPA + GW4869 group; (5) EV-OAPA + AO + GW4869 group. Briefly, BAT cells were isolated and placed in 100 nm dishes and treated with OAPA (62.5 μM), AO and/or GW4869 (10 μM). After 24 h treatment, the cell culture medium was collected then isolating extracellular vesicles (EVs) by using differential centrifugation and injected into mice by tail intravenous injection. Sting^−/−^ mice (Male, 18–22 g, 5–7 weeks old) were purchased from the Research Center of the Southern Model Organisms (Shanghai, China). In another EV injection experiment (Fig. 5J), mice were distributed into 2 groups (n = 6): (1) EV-control group; (2) EV-OAPA group. EVs from BAT cells treated with OAPA or solvent were isolated as mentioned above and then injected into Sting^−/−^ mice by tail intravenous injection. Mice were sacrificed at the end of all behavioral experiments and serum and tissues were collected for the follow-up experiments.

### Histopathology analysis

After sacrificed, liver, BAT and WAT tissues were fixed in a 4% paraformaldehyde solution. The samples were subsequently dehydrated, embedded in paraffin, and sliced into 4–4.5 μm-thick sections for Hematoxylin and Eosin (H&E) staining. For Oil Red O staining, freshly cut sections from frozen livers and BAT were washed with PBS and fixed in 4% paraformaldehyde. Subsequently, Oil-red powder was dissolved in an isopropanol solution (0.5 g/ml). After rinsing with PBS, the samples were stained with Oil Red O solution at room temperature for 30 min. Each section of the tissues was observed under a light microscope (Leica, Wetzlar, Germany), and the most representative field was selected as the final result.

### Rectal temperature measurement and infrared thermography

The mice were exposed to continuous cold stress for 5 h. The core body temperature was measured every hour using a rectal temperature probe. Prior to and after cold exposure, infrared images of the mice's shaved interscapular area were captured using a Fluke Ti400 (FLUKE, Washington, USA).

### RNA-sequencing analysis

Total RNA was extracted from mouse BAT tissues and each group contained four samples for RNA-sedanalysis. RNA purity was detected by NanoRhatometer^@^ spectrophotometer (IMPLEN, USA) and RNA integrity was assessed using the RNA Nano Assay kit. mRNA was purified from total RNA by poly-Toligo-attached magnetic beads. Following purification, the fragmented poly (A) RNA underwent reverse transcription to produce the cDNA and cDNA fragments of 250–300 bp were preferentially enriched. Sequencing library preparation was carried out using NEBNext UItraTM RNA Library Prep Kit and was further sequenced on an Illumina Novaseq platform (Illumina, USA). Gene Ontology (GO) and Kyoto Encyclopedia of Genes and Genomes (KEGG) enrichment analysis of differentially expressed genes were performed by the cluster profile R package. Based on GO and KEGG dataset, differential expression data were further analyzed by Gene Set Enrichment Analysis. Scores were obtained from TopRank and MeanRank, CHIP-X Enrichment Analysis Version 3(ChEA3) was used for the transcription factor enrichment analysis.

### Extraction and identification of mitochondrial DNA (mtDNA)

Total DNA was separated and depurated from EVs in mouse serum and cell culture medium by Gentra Puregene Cell Kit (158,745, QIAGEN) according to the manufacturer’s instruction. mtDNA was quantified by qPCR (mt-16 s/m-18 s Cyto C/rn-18 s mt-Nd3/rn-18 s) and then identified using DNA gel electrophoresis.

### Cell culture and treatment

AML12 cells (murine hepatocyte cell line) were purchased from the BNBIO biotechnology (Beijing, China) and cultured in Dulbecco’s modified Eagle medium (DMEM) supplemented with penicillin G (100 U/ml), streptomycin (100 μg/ml) and 10% fetal bovine serum (FBS) in a humidified atmosphere 5% CO_2_ at 37 °C. Mouse brown adipocytes and white adipocytes were extracted based on previous research methods [8, 14], and cultured in 10% FBS (Gibco)-supplemented DMEM/F12 medium (Gibco). Except for the first cell culture medium was replaced after 12 h, the cell culture medium was displaced every 2 days for cell growth. Upon reaching 100% confluence in a dish, pre-adipocytes were seeded in 6-well plates at the density of 5 × 10^5^ cells/well and the cells culture medium was replaced with different condition medium (DMEM)/F12 with 10% FBS, 2 μg/mL insulin, 1 nM triiodothyronine (T3), 5 mM 3-isobutyl-1-methylxanthine (IBMX), 1 μM dexamethasone, and 2 μM rosiglitazone) with or without AO (15 μM) until obtaining mature adipocytes. The applied dosage of AO was determined based on our previous [8]. Following two weeks of culturing to obtain mature adipocytes, the mature adipocytes were stimulated with OA (62.5 μM) and PA (150 μM) or AO for 24 h and collected relative conditional medium (CM). Pretreatment of GW4869 (10 μM) for 24 h or human insulin (100 nM) for 4 h before OAPA stimulation were also applied for the inhibition of EVs or the induction of proprotein convertase subtilisin/kexin type 9 (PCSK9) in BAT cells and also collected CM to further incubate with AML12 cells. In addition, at the end of treatment, the CM of BAT cells treated with OAPA or/and AO was also collected and incubated with DNase and RNase to further treat AML12 cells [18, 19].

### Immunofluorescence (IF) staining assays

Paraffin sections were immunostained after the slides had been dewaxed and rehydrated for the following detection. The BAT cells with different treatments were also fixed with 4% paraformaldehyde in PBS, and permeabilized with 0.1% Triton X-100 for 30 min. All prepared tissue or cell samples were then incubated with relative primary antibodies overnight at 4 °C. After washing with PBS, the tissues and cells were stained with anti-mouse secondary Alexa Fluor (488) or antirabbit secondary Alexa Fluor (594) antibodies for 1 h. The tissues and cells were subsequently washed and stained with DAPI for nuclear counterstaining and imaged by Olympus FV3000 confocal laser scanning microscopy (Tokyo, Japan). ROS and mito-tracker molecular probes ROS assay kit (S0033S) were obtained from Beyotime Biotechnology (Shanghai, China) and used to detect the opening of the inner mitochondrial membrane and production of ROS. After adding 10 mM of DCFH-DA probe to the cell culture and incubating it at 37 °C for 30 min, cells were then stained with DAPI, washed and imaged by Olympus FV3000 confocal laser scanning microscopy (Tokyo, Japan).

### Detection of the mPTP

Cyclosporin A (CSA, 59,865-13-3, MedChemExpress) was an mPTP de-sensitizer and effectively blocked MPTP opening by binding and blocking CypD. FCCP was an oxidative phosphorylation (OXPHOS) uncoupling agent in mitochondria and was used to establish depolarization of mitochondria membrane potential. The specific experimental steps are as follows: BAT cells were performed in the presence and absence of AO and OAPA, as well as 1 mM cyclosporine A (CsA), an inhibitor of MPTP or FCCP. At the end of treatment, the cell culture medium was discarded and cells were washed twice with PBS. Mitochondrial Permeability Transition Pore Assay Kit (C2009S, Beyotime, Shanghai, China) was used to detect the permeability transformation of the mitochondrial inner membrane following the manufacturer’s instruction. The control group was added to Calcein AM staining reagent and other experimental groups were given fluorescence quenching solution, respectively. After 30 min incubation, the cells were cleaned with PBS twice to remove excess dye and staining with DAPI. All cells were then imaged by Olympus FV3000 confocal laser scanning microscope (Tokyo, Japan).

#### Co-immunoprecipitation (Co-IP) assay

At the end of treatment, BAT adipocytes were washed with ice-cold PBS and lysed in CO-IP lysis buffer on ice. The cell lysates were centrifuged at 14,000 × g and 4 °C for 15 min. For detecting acetylation assays, the supernatants were incubated with the antibody against CypD-bound beads at 4 °C overnight. For detecting ubiquitination assays, the supernatants were incubated with the antibody against voltage-dependent anion channel 1 (VDAC1)-bound beads for an entire night at 4 °C. These beads were then resuspended, extensively washed three times with IP buffer, and subjected to SDS‒PAGE for immunoblotting analysis with anti-Histone H3 (acetyl K27) or anti-Ubiquitin (linkage-specific K63) antibodies, respectively.

#### Transmission electron microscopy (TEM)

Cubic BAT pieces were fixed with 2.5% glutaraldehyde diluted in sodium phosphate (0.1 M, pH 7.4) for 24 h at 4 °C. Tissue samples were dehydrated through a graded alcohol series and embedded in Epon Araldite after postfixation in 1% OsO4 for 1 h. Ultrathin Sects. (50 nm) were produced using an ultramicrotome (Leica, Wetzlar, Germany) before staining with uranyl acetate and lead citrate. The specimens were visualized using an electron microscope. Images were taken using an FEI Tecnai G2 Spirit transmission electron microscope (Hillsboro, OR, USA).

#### Statistical analysis

All results were repeated at least three independent times and normal distribution variables were expressed as mean ± SEM. Comparison of data was performed using a one-way ANOVA and Tukey’s post-hoc test between multiple groups in GraphPad Prism Software 9.0 (GraphPad, San Diego, CA, USA). Statistical significance was determined as *p < 0.05, **p < 0.01, and ***p < 0.001.

Additional details about used antibodies and methodology employed in this study were provided in the supplementary files.

### Results

#### AO increases BAT thermogenesis and relieves liver inflammation in obese mice

To further verify the effects of AO on BAT and liver, we established a mouse model fed with an HFHS diet and treated with AO as shown in Fig. 1A. Compared with those receiving the vehicle treatment, both normal and obese mice administered with AO were obviously protected from weight gain, without the changes of caloric intake (Fig. S1A and S1B). Notably, we observed an increase in the weight of BAT (Fig. 1B) but not liver (Fig. 1C and Fig. S1C) following AO treatment either under a normal diet or HFHS diet, suggesting that AO was able to simulate the proliferation of BAT cells. On the contrary, the weight of WAT was decreased under the same condition (Fig. S1D and S1E). The size of lipid droplets in adipocytes serves as an indicator of lipid consumption and accumulation. Pathological examination revealed that AO reversed the whitening of BAT and the enlargement of lipid droplet size in WAT, and improved hepatic steatosis in HFHS diet-fed mice. Meanwhile, AO reduced the lipid droplet size in the BAT in HFHS diet-fed mice (Fig. 1D, E and Fig. S1F). Acute cold exposure significantly increases systemic energy expenditure and is beneficial to observe the regulatory effects of medicines on BAT thermogenesis. To confirm the changes in BAT thermogenesis in response to AO treatment, we placed the mice under continuous cold stress (4 °C) for 5 h and measured the core body temperature every hour using a rectal temperature probe (Fig. 1F and Fig. S1G). Obviously, the body temperature of mice in the AO administration groups declined slower than that in the chow diet group or HFHS group. We previously found that AO increased oxygen consumption, carbon dioxide excretion and heat production in the dark cycle [8]. Collectively, we speculated that AO promoted thermogenesis in BAT after cold stimulation. Based on these changes, we further measured the levels of total triglyceride (TG), total cholesterol (TC), alanine aminotransferase (ALT), aspartate aminotransferase (AST) and nonesterified free fatty acids (NEFA) levels in serum or different tissues. After 4 weeks of administration, AO markedly decreased the HFHS diet-induced serum levels of AST, ALT, NFEA, TG and TC, and hepatic levels of NEFA, TG and TC but showed less effects on these parameters in BAT (Fig. S1H-S1J). We also examined the intrahepatic activation of cyclic GMP-AMP synthase (cGAS)/ STING pathways, a pronounced inflammatory pathway found in fatty liver diseases [20]. Notably, the mRNA and protein levels of STING, cGAS, TBK1, NF-ĸB, IL-1β, IL-6 and TNFα, as well as the phosphorylation of TBK-1, were all markedly increased in the HFHS group but were effectively alleviated after AO treatment (Fig. 1G, H and Fig. S1K).

### AO treatment in donor mice protects obese mice-derived BAT-induced hepatic inflammation in chow diet-fed recipient mice.

We previously demonstrated that the ablation of BAT suppressed the protective and lipid-lowering effects of AO on the liver. To further explore the pathological intercellular communication between BAT and liver, donor mice were fed an HFHS diet with or without the oral administration of AO. Subsequently, chow diet-fed mice were subjected to interscapular BAT resection, and the BATs derived from donor mice fed with HFHS diet were transplanted into the dorsal abdominal region of the recipient mice (Fig. 2A). Meanwhile, recipient mice in the sham group were transplanted with BAT isolated from donor mice with fed chow diet to avoid graft rejection. Although the transplantation of different BAT types did not affect the appearance or weight of the liver (Fig. 2B), significant inflammation and slight necrosis were observed in mice that received BAT derived from obese mice, namely BAT (HFHS), but not in mice received BAT derived from AO-treated obese mice, referred as BAT (AO-HFHS) (Fig. 2C and D). Interestingly, mice transplanted with BAT (AO-HFHS) maintained a higher core body temperature after acute cold stress stimulation compared to those mice transplanted with BAT (HFHS) (Fig. 2E). As shown in Fig. 2F and Fig. S2A, the serum levels of AST, ALT, NEFA were increased in mice received BAT (AO-HFHS) instead of mice received BAT (AO-HFHS). Consistent with these indications of liver injury, the transplantation of BAT (HFHS) dramatically elevated the mRNA levels of *Sting, Cgas, Il1b, Il6* and *Tnfa* in livers and protein levels of the cGAS-STING pathway and its downstream targets including P-TBK1, TBK1, NF-ĸB, IL-6 and IL-1β in the recipient mice, of which changes were not observed in the mice transplanted with BAT (AO-HFHS) (Fig. 2G, H and Fig. S2B).

### The transplantation of BAT derived from AO-treated lean mice protects HFHS-induced obesity and hepatic inflammation in recipient mice

To further confirm that the hepatoprotective effect of AO is entirely caused by BAT transplantation, recipient mice were fed with an HFHS diet while simultaneously transplanting BAT from control or AO-treated chow diet-fed mice, referred as BAT (Ct) or BAT (AO), respectively (Fig. 3A). The transplantation of BAT (AO) lowered the bodyweight of recipient mice fed with the HFHS diet at a certain degree (Fig. 3B). Although the weight gain and lipid droplet accumulation were found in the transplanted BAT (Ct) in recipient obese mice, the transplanted BAT (AO) tissues maintained their original morphology BAT (AO) markedly improved the enlargement of adipocytes in WAT and further protected liver vacuolation and intrahepatic infiltration of inflammatory cells in the recipient mice fed with HFHS diet (Fig. 3C–F, Fig. S3A and S3B). According to infrared image analysis, the interscapular BAT skin temperature and core body temperature of obese mice were largely increased after transplanted with BAT (AO), compared with transplanted with BAT(Ct) (Fig. 3G). Correspondingly, serum and hepatic levels of ALT and AST, serum levels of TG, TC and NEFA, and BAT NEFA levels in HFHS mice transplanted with BAT (AO) showed a decreasing trend when compared to obese mice transplanted with BAT (Ct) (Fig. 3H and Fig. S3C). As expected, the transplantation of BAT (AO) also decreased the mRNA expression of *Sting, Cgas, Il1b, Il6* and *Tnfa* and the activation of the cGAS-STING pathway as illustrated in Fig. 3I, J and Fig. S3D. These results suggest that BAT treated with AO can maintain a therapeutic phenotype, highlighting the critical role of BAT in the protective effects of AO against obesity and liver inflammation.

We also investigated whether the protective effects of AO on obese mice were also related to WAT function. Fig. S4A and S4B depicted the animal experiment procedures, dosing regimens and the changes of body weight in recipient mice. As demonstrated in Fig. S4C and S4D, the transplantation of WAT derived from AO-treated lean mice, referred as WAT (AO), slightly increased the weight and volume of BAT, while had minimal effects on the lipid droplet size in WAT or BAT and the livers of recipient obese mice (Fig. S4E and S4F). Additionally, there was no significant difference in thermal imaging data or rectal temperature before or after WAT transplantation (Fig. S4G). Furthermore, the transplantation of WAT (AO) did not affect the activation of cGAS-STING pathway or its downstream targets, nor influence WAT lipid metabolism in the obese mice (Fig. S4H, S4I and S4J). These results underscore the significance of a potential communication networks between the liver and BAT, rather than WAT, in the overall lipid-lowering effects of AO.

### AO inhibits excessive oxidative stress and mtDNA leakage in BAT

**Fig. 4:**
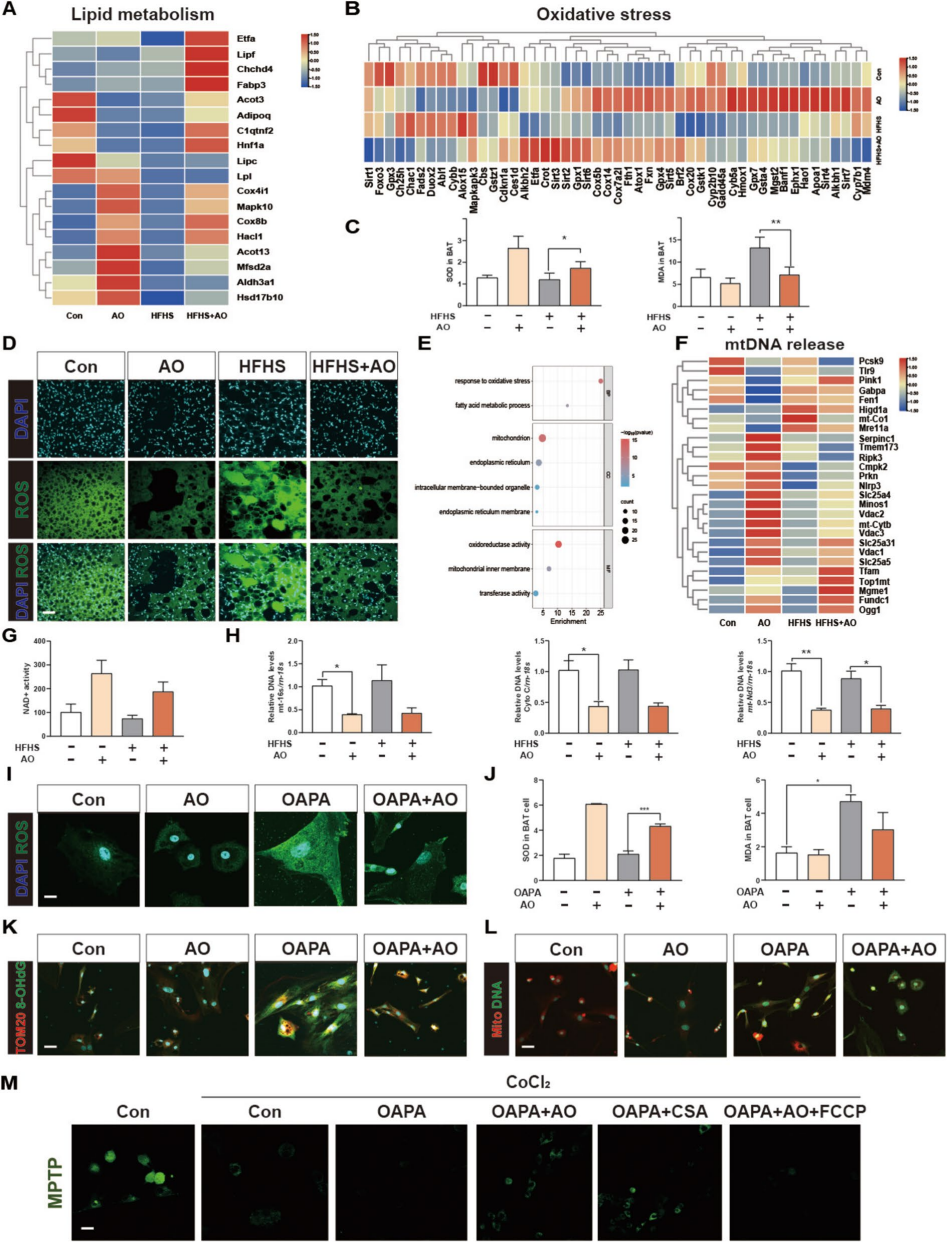
AO improves oxidative stress and reduces the release of mtDNA in BAT. The relative expression levels of differentially expressed genes implicated in lipid metabolism **A** and oxidative stress **B** were shown as a heatmap. **C** The levels of SOD and MDA in BAT of mice. **D** Representative images of immunofluorescence staining for ROS and DAPI of brown adipose tissues. Scale bar = 40 μm. **E** Analysis of GO pathway enrichment. Bubble Diagram was used to display the relative expression levels of genes that were closely related to mitochondrial function. **F** A heatmap displayed genes involved in the release of mtDNA. **G** The level of NAD.^+^ activity in BAT of mice. **H** Relative mtDNA levels of *mt-16 s, Cyto C* and *mt-Nd3* in serum. *Rn-18 s* was used as an internal reference. **I** Representative ROS/DAPI staining images of BAT primary cells. Scale bar = 40 μm. **J** The levels of SOD and MDA in BAT primary cells. Representative **K** TOM20 (red)/8-OhdG (green) or **L** Mito Tracker (red)/DNA (green) with DAPI (blue) staining images of the BAT primary cells. Scale bar = 100 μm. **M** MPTP assay kit was used to detect the permeability transformation of mitochondrial inner membrane following the manufacturer's instruction in BAT primary cells. The cells were given AO, OAPA, CSA and FCCP for experimental comparison. Scale bar = 100 μm. Statistical significance: *P < 0.05, **P < 0.01, ***P < 0.001, compared between groups (n = 6 for mice and n = 3 for cell experiments)

In-depth analysis of RNA sequencing data was performed for AO-treated BAT and results are shown as a clustered heatmap for mass DEGs. As demonstrated in Fig. 4A and B, AO significantly increased the expression of genes involved in lipid metabolism, such as acyl-CoA thioesterase 13 (*Acot13*), cytochrome C oxidase subunit 8b (*Cox8b*), cytochrome C oxidase subunit IV (*Cox4il*), 2-Hydroxyacyl-coA lyase 1 (*Hacl1*) and mitogen-activated protein kinase 10 (*Mapk10*). Furthermore, genes about antioxidant stress like sirtuin3 (*Sirt3*), cytochrome P450 2b10 (*Cyp2b10*), *Cyp7b1*, ferritin heavy chain 1 (*Fth1*) and frataxin (*Fxn*) were found to be upregulated. In contrast, genes implicated in oxidative stress like cytochrome b-245 Beta Chain (*Cybb*), *Abl1*, dual oxidase 2 (*Duox2*), fatty acid desaturate 2 (*Fads2*) and chac glutathione specific gamma-glutamylcyclotransferase 1 (*Chac1*) exhibited decreased expression following AO treatment. Consequently, we employed enzymatic methods to measure superoxide dismutase (SOD) and malondialdehyde (MDA) concentrations in the BAT tissue of mice to assess oxidative stress levels. AO increased the SOD levels but decreased MDA levels in the BAT of obese mice (Fig. 4C). Concurrently, fluorescence microscopy using DCFH-DA staining indicated obvious lower fluorescence signals in BAT from mice treated with AO, indicating decreased ROS levels in these mice (Fig. 4D). Particularly, KEGG analysis further revealed considerable gene enrichment in response to oxidative stress, fatty acid metabolic processes, and pathways related to mitochondria and lipid oxidation (Fig. 4E). Given that mitochondria are the key organelles responsible for enhanced biological activities, we further used sequencing data to investigate the transcription of genes related to mitochondrial structure, function and morphology. Some studies have pointed out that mtDNA binding enlarges the pore of VDAC1, and the opening of mPTP is essential for the release of damaged mtDNA from the nucleoid to the intermembrane space [21]. Conversely, mitophagy plays a crucial role in removing damaged mitochondria during cytoplasmic remodeling and breaks down damaged and mutant mtDNA, which improves mitochondrial malfunction and illness [22]. Notably, AO significantly inhibited the release of mtDNA by regulating kexin type9 (*Pcsk9*)*,* solute carrier family 25 member 4 (*Slc25a4*), *Slc25a5*, *Vdac1* and *Vdac2* targets (Fig. 4F). We also found a marked increase of NAD^+^ after administration, and NAD^+^ content is inversely related to mPTP opening (Fig. 4G). Furthermore, the circulating mtDNA levels in mouse serum were also investigated. Serum mtDNA levels (determined by analyzing three typical mitochondrial genes including mt*−16 s, Cyto C* and *mt-Nd3*) were significantly decreased after AO treatment (Fig. 4H). Additionally, we employed OAPA to establish a lipid accumulation model in BAT cells in vitro and examined the pharmacodynamic effect of AO. Consistently, in OAPA-treated BAT cells, we observed increased SOD activity along with decreased MDA and ROS signals (Fig. 4I, J and Fig. S5A). Besides, OAPA significantly raised the content of oxidized DNA and cytosolic mtDNA levels in BAT cells, which were all reversed after AO administration (Fig. 4K, L, Fig. S5B and S5C). CsA, a known mPTP inhibitor, significantly increased Calcein fluorescence compared to the OAPA group. Additionally, FCCP was shown to induce mPTP opening and prevent the rise in Calcein fluorescence caused by AO treatment (Fig. 4M). These findings suggest that AO can mitigate oxidative stress and inhibit mPTP opening, thereby reducing the release of mitochondria-related lipotoxic substances in BAT.

### AO protects overwhelmed BAT from releasing DNA-enriched EVs to improve STING-dependent hepatic inflammation

**Fig. 5:**
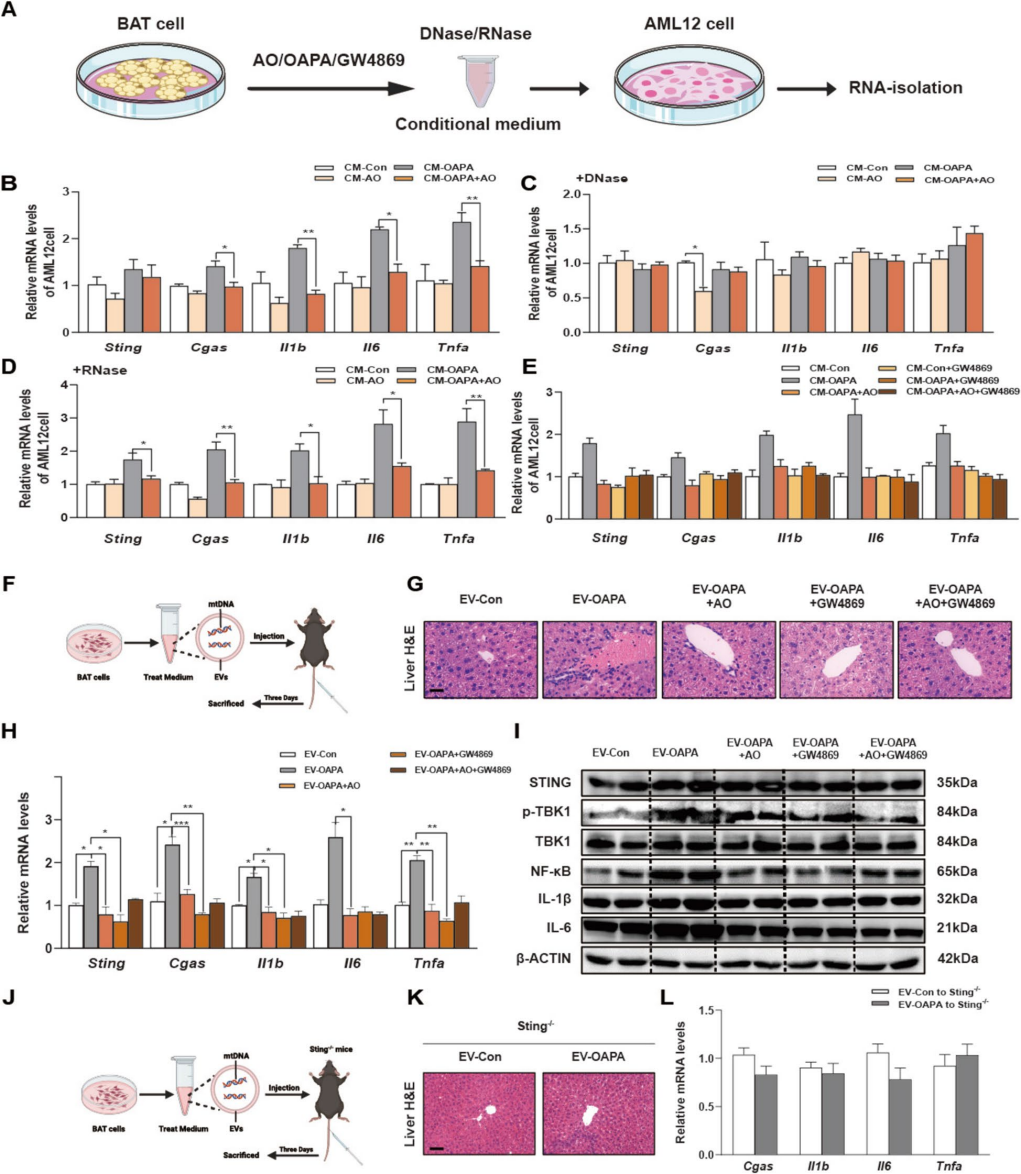
AO protects overwhelmed BAT from releasing DNA-enriched EVs to improve STING-dependent hepatic inflammation. **A** The BAT cells were treated with OAPA and/or AO with or without the presence of GW4869 according to experimental methods for CM collection. The CM was also either incubated with DNase or RNase. All above different CM were given to AML12 cells for 24 h. **B**–**E** The relative mRNA levels of *Sting*, *Cgas*, *Il1β*, *Il6* and *Tnfα* by qPCR and *Hprt1* was used as an internal reference. **F** The BAT cells were treated with different concentrations of AO, OAPA, with or without GW4869 for CM collection. These CM were then given mice through a tail vein and sacrificed after 3 days. **G** H & E images of liver tissues of the mice. Scale bar = 20 μm. **H** Relative mRNA levels of *Sting*, *Cgas*, *Il1β*, *Il6* and *Tnfa* were detected by qPCR, using *Hprt1* as an internal reference in the liver tissues of the mice. **I** Protein levels of STING, P-TBK1, TBK1, NF-ĸB, IL-1β and IL-6 were tested by western blot analysis and normalized with β-ACTIN. **J** The BAT cells were treated with OAPA or solvent for CM collection, which were then given to Sting^−/−^ mice through a tail vein and sacrificed after 3 days. **K** H & E images of liver tissues of the Sting.^−/−^ mice. Scale bar = 20 μm. **L** Relative mRNA levels of *Sting*, *Cgas*, *Il1β*, *Il6* and *Tnfa* were detected by qPCR, using *Hprt1* as an internal reference in liver tissues of the mice. Statistical significance: *P < 0.05, ** P < 0.01, ***P < 0.001, compared between groups (n = 6)

Next, we aimed to investigate whether overwhelmed BAT activates liver inflammation through the leakage of mtDNA, and determine the underlying mechanism of this transmission (Fig. 5A). Briefly, primary BAT cells were treated with OAPA and/or AO, and conditional medium (CM) was collected and subsequently used to cultivate AML12 cells. Interestingly, CM derived from OAPA-treated BAT cells significantly induced inflammation in AML12 cells, as evidenced by the activation of cGAS-STING pathway and increased expression of *Il1b, Il6* and *Tnfa*. On the other hand, AO protected BAT cells from OAPA-induced injury and CM derived from these cells had no effects on the expression of proinflammatory genes (Fig. 5B). We then applied DNase or RNase to eliminate leaked DNA or RNA in these OAPA- or AO-treated CM and further incubated with AML12 cells at 37 °C for 24 h. As expected, the presence of DNase but not RNase completely abrogated CM-triggered activation of inflammatory STING signaling in hepatocytes, as evidenced by decreased mRNA levels of *Sting, Cgas, Il1b, Il6* and *Tnfa* in AML12 cells (Fig. 5C, D). This also confirms that BAT transferred DNA (most likely mtDNA) rather than other exogenous factors like OAPA after being stimulated by high fat. Furthermore, we successfully extracted mtDNA from culture medium of OAPA- or AO-treated BAT cells. The mtDNA levels in CM (determined by analyzing three typical mitochondrial genes including mt-16 s, Cyto C and mt-Nd3) were significantly decreased after AO treatment (Fig. S6A). Additionally, we incubated mouse hepatocytes with CM collected from OAPA- and AO-treated WAT adipocytes as well as WAT CM treated with DNase and RNase. However, CM derived from overwhelmed WAT only slightly induced inflammation in AML12 cells, and the removal of either DNA or RNA from the WAT CM failed to improve the inflammatory responses in hepatocytes (Fig. S6B-D).

Depending on the severity of mitochondrial damage [23], mitochondria-derived EVs can carry materials that act as damage-associated molecular patterns (DAMPs) and then initiate sterile inflammation in recipient cells or tissues. We then investigated whether the transfer of damage-associated DNA from overwhelmed BAT to liver relies on EVs. By using a specific EV inhibitor GW4869, we found that the inhibition of EVs formation and secretion in BAT cells completely blocked the stimulation of hepatic STING pathway and related inflammatory responses induced by CM derived from OAPA-treated BAT cells (Fig. 5E). To verify the extraction of EVs, we detected specific markers HSP70, flotillin-1, CD63, CD9 and calnexin by using WB. The result proved that the EVs were successfully extracted from the CM of BAT cells (Fig. S6E). Furthermore, we isolated and purified EVs from control, OAPA-, and OAPA + AO-treated BAT cells and injected these EVs into mice by tail vein injection (Fig. 5F). The H&E staining of liver sections of the recipient mice further supported our findings that DNA enriched EVs derived from overwhelmed BAT could trigger the intrahepatic infiltration of inflammatory cell. However, EVs derived from AO- or GW4869-treated overwhelmed BAT cells had no effects on hepatic inflammation (Fig. 5G). These findings were further supported by qPCR and WB analysis (Fig. 5H, I and Fig. S6F).

We further validated whether DNA serves as the carrier for the transmission of damage signals between BAT and the liver in vivo by utilizing STING^−/−^ mice that are incapable of eliciting DNA-mediated inflammatory responses. As shown in Fig. S6G, STING was effectively knocked down in the livers. As anticipated and in lined with the in vitro findings, the transplantation of DNA-enriched EVs derived from overwhelmed BAT cells via tail vein injection failed to induce significant inflammation in the recipient STING^−/−^ mice (Fig. 5J). This lack of inflammation was corroborated by histological examination using H&E staining, as well as qPCR, and WB analysis (Fig. 5K, L and Fig. S6H). These results suggest that damage-associated DNA derived from overwhelmed BATs can be transported to the liver by EVs, ultimately triggering STING-dependent hepatic inflammation.

### AO blocks the opening of the mPTP by decreasing the acetylation of CypD and inhibiting ANT function

**Fig. 6:**
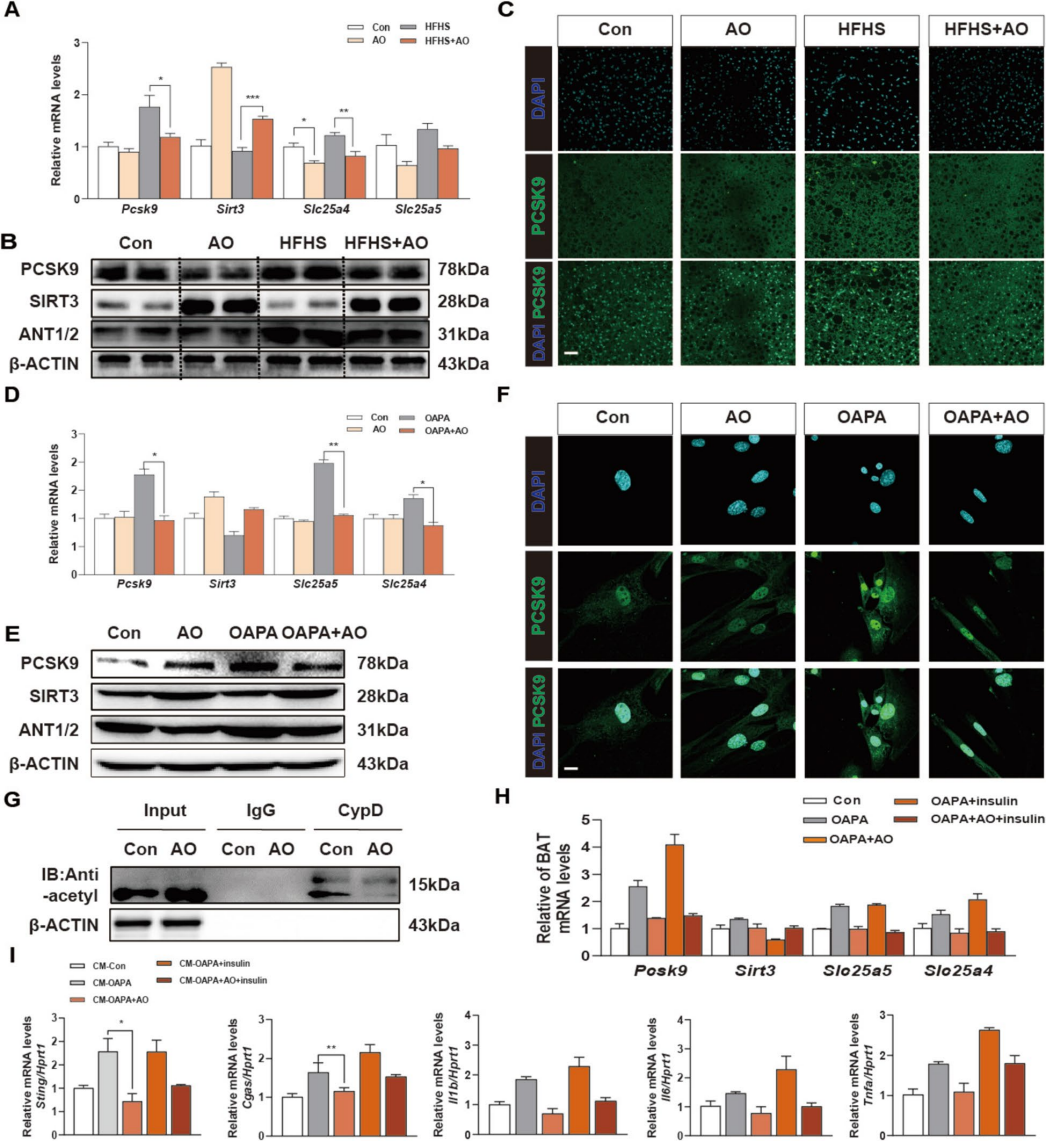
AO decreases the acetylation of CypD and ANT function to inhibit mPTP opening in BAT cells. **A** Qualified relative mRNA levels of *Pcsk9*, *Sirt3*, *Slc25a4* and *Slc25a5* were determined by qPCR and normalized using *Hprt1* as an internal control in mouse BAT. **B** Representative immunoblots of PCSK9, SIRT3, ANT1/2 in mouse BAT and β-ACTIN was used as the loading control. **C** Representative PCSK9 and DAPI staining images of BAT. **D** The relative mRNA levels of *Pcsk9*, *Sirt3*, *Slc25a4* and *Slc25a5* were determined by qPCR, and *Hprt1* as an internal control in BAT cells. **E** Protein levels of PCSK9, SIRT3, ANT1/2 were detected by western blot analysis and normalized with β-ACTIN. **F** Representative PCSK9 and DAPI staining images of BAT cells. **G** Co-IP experiments in BAT cells. **H** The BAT cells were treated with AO and OAPA, with or without insulin for RNA extraction and CM collection. The cell mediums were then given to AML12 cells for 24 h and extracted RNA. Relative mRNA levels of *Pcsk9*, *Sirt3*, *Slc25a4* and *Slc25a5* in BAT cells were determined by qPCR. **I** Relative mRNA levels of *Sting*, *Cgas*, *Il1β*, *Il6* and *Tnfa* in AML12 cells. *Hprt1* was used as an internal control. Statistical significance: *P < 0.05, ** P < 0.01, ***P < 0.001, compared between groups (n = 6 for mice samples and n = 3 for cell experiments)

Under oxidative stress, mtDNA can be released into the cytoplasm or secreted to the extracellular spaces due to a change in MMP and subsequent opening of mPTP [24]. The formation and opening of mPTP rely on a large conductance pore-forming complex, composed of CA located at the outer mitochondrial membrane, ANT located at the inner mitochondrial membrane and CypD in the matrix [25, 26]. Recent studies have identified several novel lipid biomarkers in obesity, with a focus on PCSK9 as a key molecule regulating lipid metabolism. The association between PCSK9 and mPTP components, including VDAC and ANT, has been highlighted to be essential for the downstream opening of mPTP, DNA leakage and following STING-dependent inflammation [27]. Furthermore, a negative correlation between the expression of PCSK9 protein and SIRT3 has been found and the inhibition of SIRT3 eliminated the effects of PCSK9 on inflammation and mitochondrial ROS overload [28]. Notably, AO significantly counteracted the stimulatory effects of the HFHS diet on the transcription and translation of genes associated with PCSK9, SIRT3, ANT, *Slc25a4* and *Slc25a5* in the mouse BAT (Fig. 6A, B and Fig. S7A). Furthermore, immunofluorescence staining results confirmed that AO decreased the expression and of PCSK9 in the BAT when compared with the HFHS-fed mice (Fig. 6C). Consistently, we also confirmed the inhibitory effects of AO on the expression of mPTP opening-related genes (Fig. 6D, E and Fig. S7B). Similarly, AO reduced the upregulation of PCSK9 induced by OAPA in primary BAT cells (Fig. 6F). Furthermore, it has been reported that mPTP is inhibited by the deacetylation of CypD in a SIRT3-dependent manner [29]. As depicted in Fig. 6G, IP experiments found that AO significantly promoted the deacetylation of CypD in BAT cells. We then utilized insulin to directly induce PCSK9 expression in primary BAT cells. The induction of *Pcsk9* synergized with OAPA in suppressing the expression of *Sirt3* while promoting the expression of downstream genes including *Slc25a4* and *Slc25a5* in BAT cells, and these effects were almost completely blunted by AO treatment (Fig. 6H). Meanwhile, CM derived from BAT cells treated with OAPA and insulin significantly activated STING-related inflammatory responses in the hepatocytes, which were all remarkably reversed by AO treatment in BAT cells (Fig. 6I). These results indicated that PCSK9-SIRT3-CyD axis are potential targets of AO treatment in protecting mPTP opening and DNA leakage in overwhelmed BAT cells.

### AO prevents the opening of the mPTP by promoting PTEN-induced putative kinase 1 (PINK1)/parkin-mediated mitophagy and inhibiting VDAC1 oligomerization

**Fig. 7:**
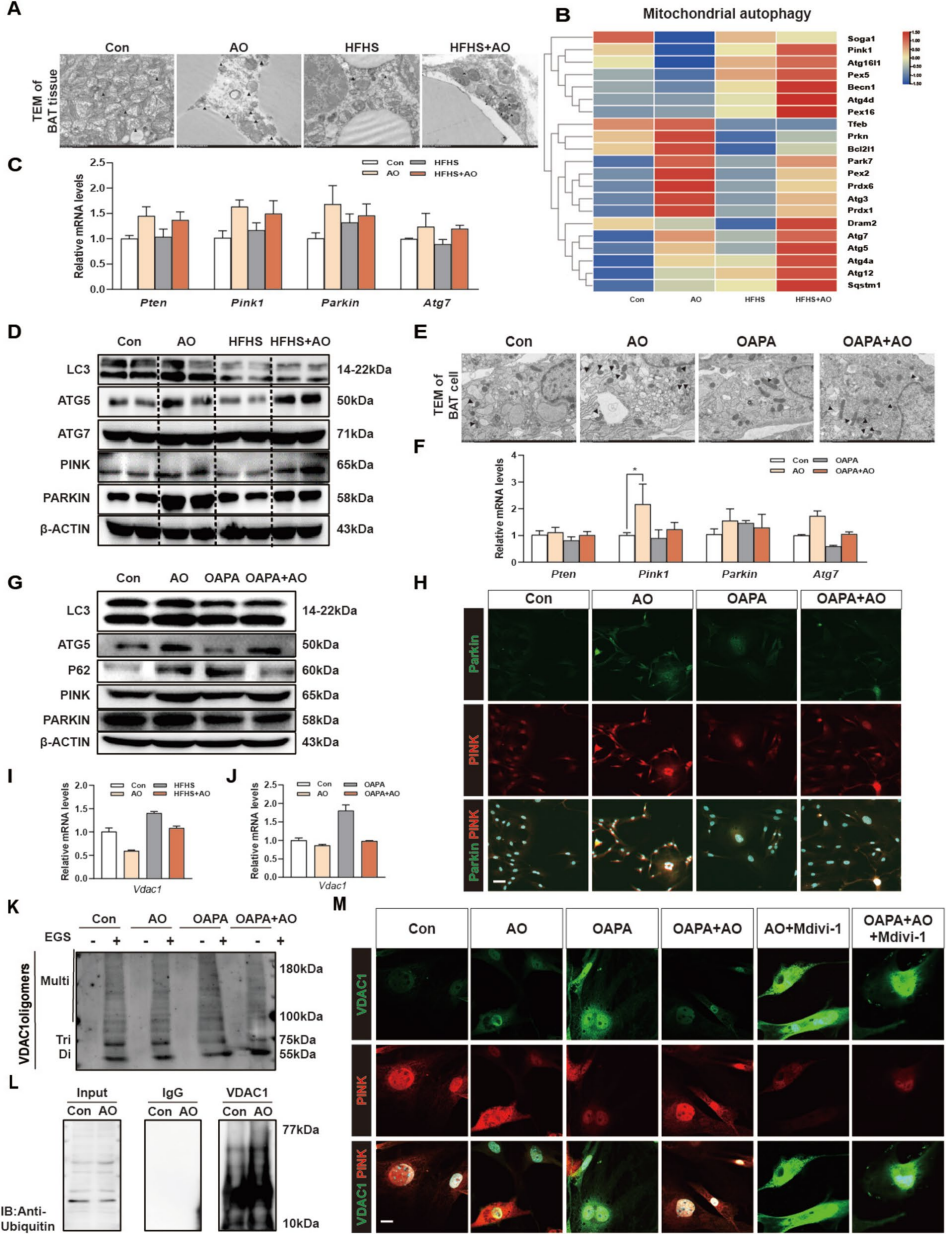
AO promotes mitochondrial autophagy and inhibits VDAC1 oligomerization. **A** Transmission electron microscopy image of the BAT in mouse. Arrow marks the mitochondrial autophagy situation. Scale bar = 2 μm. **B** The relative expression levels of differentially expressed genes implicated in mitochondrial autophagy was shown as a heatmap. **C** The relative mRNA levels of *Pten*, *Pink1*, *Parkin*, and *atg7* were detected by qPCR and normalized with *Hprt1* in BAT. **D** Representative immunoblots of LC3, Atg5, Atg7, PINK, Parkin were detected by western blot analysis and β-ACTIN was used as the loading control. **E** Transmission electron microscopy image of the BAT cells. Arrow marks the mitochondrial autophagy situation. Scale bar = 2 μm. **F** Relative mRNA levels of *Pten*, *Pink1*, *Parkin*, and *atg7* were determined d by qPCR and normalized with *Hprt1* in BAT cells. **G** Protein levels of LC3, Atg5, p62, PINK, Parkin were detected by western blot analysis and β-ACTIN was used as the loading control in BAT cells. **H** Representative images of immunofluorescent co-staining of Parkin and PINK in BAT cells. The relative mRNA levels of Vdac1 were detected by qPCR and normalized with *Hprt1* in **I** BAT and **J** BAT cells. **K** Representative immunoblots of VDAC1 oligomers in BAT cells. The crosslinking reagent EGS was used to stabilize the oligomers during electrophoresis. **L** Immunoblotting of ubiquitinated VDAC1 in BAT cells. **M** Representative VDAC1, PINK and DAPI staining images of BAT cells after mdivi-1 treatment (10 μM). Statistical significance: *P < 0.05, ** P < 0.01, ***P < 0.001, compared between groups (n = 6 for mice samples and n = 3 for cell experiments)

Previous studies have pointed out that the inhibition of PCSK9 in hepatocytes reduced mitochondrial damage and the consequent inflammatory response via boosting PINK1-Parkin-mediated mitophagy [30]. Based on the rise in autophagosome/autolysosome-engulfed mitochondria in TEM, we confirmed that AO significantly boosted mitophagy in BATs in vivo (Fig. 7A). Our transcriptomic results of BAT samples also revealed higher expression of mitophagy-related genes like *Pink1*, *Atg5*, *Atg7* and *Atg3* (Fig. 7B). It has been reported that PCSK9 could interact with PTEN and cause its degradation via the lysosomal route [30], subsequently inducing PINK1/Parkin-mediated mitophagy [31]. Interestingly, by suppressing PCSK9, AO increased the mRNA levels of *Pten*, *Pink*, *Parkin*, *Atg7*, *Fundc1* and *Gpx1*, and the protein levels of PINK/Parkin and other classic mitophagy-related targets like LC3II/I, ATG5 and ATG7, accompanied with decreased P62 levels (Fig. 7C, D and Fig. S8A, S8B), suggesting the activation of mitophagy in BATs in the HFHS-fed + AO-treated mice than that in the HFHS-fed mice. We also observed similar results about the improved mitochondrial structure and morphology, and activated mitophagy in primary BAT cells treated with AO, when compared with OAPA-challenged BAT cells (Fig. 7E–H, Fig. S8C and S8D). However, these effects of AO were not observed in WAT tissues or primary WAT cells (Fig. S8E and S8F). The above-mentioned findings revealed that AO may encourage the elimination of damaged mitochondria by activating the mitophagy process.

Our experiments showed that AO upregulated the expression of Parkin. Several studies have pointed out that PARKIN-mediated ubiquitination of VDAC1 at a specific location improves liver fibrosis by disrupting VDAC1 oligomerization and mtDNA release, suggesting another potential mechanism by which AO-upregulated PARKIN protects against mitochondrial damages in overwhelmed BATs [32]. As expected, AO decreased the mRNA and protein levels of VDAC1 under lipid stimulation both in in vitro and in vivo (Fig. 7I, J and Fig. S8G, S8H). We further examine the oligomerization status of VDAC1 following treatment with the cross-linking reagent EGS. Interestingly, BAT cells exposed to OAPA showed higher levels of VDAC1 oligomers than those treated with AO (Fig. 7K). Our findings also indicated that VDAC1 was more ubiquitinated in the BAT following AO treatment (Fig. 7L). Nonetheless, results in Fig. 7M revealed that the regulative effects of AO on both PINK and VDAC1 were largely diminished by mdivi-1 (a recognized inhibitor of mitochondrial fission). These findings indicate that AO protects the mitochondrial homeostasis and inhibits the mtDNA release not only by regulating the PCSK9-SIRT3-CyD axis but also by promoting PINK/PARKIN-mediated mitophagy and PARKIN-related inhibition of VDAC oligomerization.

## Discussion

Obesity has reached epidemic proportions and is now the leading preventable cause of death worldwide. Over the past decade, research on obesity has experienced exponential growth, resulting in significant breakthroughs in body weight management. Unfortunately, during this same period, the prevalence of obesity has alarmingly increased, rendering it a major health concern in whole world. Consequently, there remains a pressing need for effective and safe pharmaceutical treatments to enhance energy consumption and manage obesity. In this current study, our findings suggested that AO effectively improved lipid accumulation and possessed anti-obesity effects both in vivo in the HFHS-induced mouse model and in vitro in the primary BAT adipocytes and hepatocytes. Interestingly, AO protected the mitochondrial homeostasis by decreasing CypD acetylation-related ANT, promoting PINK/parkin-mediated mitophagy and inhibiting VDAC1 oligomerization. Consequently, AO blocked the opening of the mPTP and the release of mtDNA from BAT to livers in EVs manner and subsequent hepatic activation of STING signaling. Our research offers a novel approach to obesity treatment by targeting the pathological communication between BAT and the liver.

Increasing energy expenditure through the stimulation of heat-generation BAT presents a promising strategy for weight loss and the treatment of obesity-related metabolic syndromes. By applying RNA sequencing analysis, we determined the molecular mechanisms by which AO alleviated obesity and regulated BAT function. Based on the outcomes of the gene sequencing analysis, we discovered that the most significant DEGs were lipid metabolism and oxidative stress genes. An imbalance in energy consumption usually leads to obesity that further promotes the induction of oxidative stress. Furthermore, increased oxidative stress may result in a positive feedback loop, which can exacerbate mitochondrial dysfunction and cause greater ROS accumulation [33]. We then investigated the activities of SOD, MDA and ROS and demonstrated that AO could possess antioxidant activity and dramatically reduce oxidative stress levels in BATs. Because of the high tendency for aberrant release of free electrons, mitochondrial OXPHOS is a primary site for the formation of ROS [34]. Due to its proximity to mtROS and the absence of repair mechanisms, mtDNA is more vulnerable and susceptible to the effects of oxidative stress compared to nuclear DNA (nDNA). When ROS concentrations reach a critical threshold, oxidative stress can damage the mitochondrial membrane, leading to the release of mtDNA from malfunctioning mitochondria into the cytosol [35]. BAT contains a high concentration of mitochondria and is involved in energy expenditure, thermogenesis and glucose homeostasis. Our gene expression analysis suggests that AO may operate as a natural regulator of mtDNA, which repairs the function of mitochondria and regulates downstream mitochondria-related genes by inhibiting the formation of oxidized mtDNA and the leakage of mtDNA into the cytoplasm, observed in both primary BAT adipocytes and BATs from obese mice (Fig. 4).

Data obtained from primary BAT cells provided direct evidence that AO has a positive impact on reducing MMP and discouraging mPTP opening (Fig. 4). We also investigated whether AO could block the release of mtDNA from BAT mitochondria into the cytoplasm. SIRT3 has the ability to deacetylate CypD, which is a critical component of the mPTP that avoids mitochondrial malfunction and inhibits the opening of mPTP [36–38]. We demonstrated that AO treatment markedly increased the level of SIRT3 and decreased the acetylation of CypD in BAT cells since its deacetylation decreases the likelihood of ANT-mediated mPTP opening (Fig. 6). The VDAC1 is a crucial channel located in the outer mitochondrial membrane that facilitates the movement of small molecules and ions between the mitochondrial intermembranous region and the cytoplasm. Together with the ANT in the inner mitochondrial membrane, VDAC is believed to form the core of a mitochondrial multiprotein complex called the mPTP. PINK1-Parkin-mediated mitophagy is one of the most well-known mitophagy routes. Parkin-induced ubiquitination of VDAC1 was recently discovered, indicating that VDAC1 ubiquitination may modulate mitochondrial phenotypes following Parkin activation [39]. Also, our findings suggest that AO activated PINK-Parkin and ubiquitination of VDAC1 K53 and inhibited VDAC1 oligomerization to inhibit the mPTP opening and VDAC1's ability for liberating mtDNA from primary BAT adipocytes and BATs of obese mice. Notably, the regulatory effects of AO on these two targets of ANT and VDAC1 and subsequent inhibition on mPTP were achieved by inhibiting their common target, PCSK9 (Figs. 6 and 7). Recent research has established a link between low levels of PCSK9 and condition such as metabolic syndrome, obesity, insulin resistance and diabetes [40].

We previously demonstrated that AO increased autophagy flux and improved liver steatosis and decreased the expression of targets involved in lipid biosynthesis [14]. Furthermore, AO enhanced systemic fat consumption by promoting thermogenesis mediated by PPAR-UCP1, ultimately leading to a reduction in obesity through improved BAT function [8]. Here, we found that AO not only notably increased BAT weight, volume and heat production in fatty mice, but also mitigated the abnormal increase in liver inflammatory cell proliferation and production of inflammatory mediators caused by lipid accumulation, suggesting a significant alleviation of local liver inflammation (Fig. 1). However, after BAT excision, PPARα and downstream gene expression in the liver were not increased by AO anymore in previous research [8]. Recent studies have demonstrated that the BAT can produce several proteins or DAMPs, which are secreted into the bloodstream and play a role in regulating oxidant balance and systemic glucose and lipid metabolism [32]. We have initially concluded that AO not only has the potential to activate BAT and further alleviate obesity, but also affect the communication between BAT and liver in obesity. As a consequence, we investigated whether livers of normal mice were affected after BAT transplantation from obese mice. BAT transplantation from obese mice led to elevated the mRNA levels of inflammatory cytokines in the livers of normal mice by activating the cGAS/STING pathway (Fig. 2). Moreover, we demonstrated that transplantation of BAT under AO stimulation promoted thermogenesis in recipient mice and markedly decreased the mRNA and protein levels of inflammatory factors in obese mice (Fig. 3). Although AO can stimulate the browning of WAT in cold environments, the transplantation of WAT with AO treatment had little effect on the thermogenesis and inflammatory cytokines production of the liver in obese mice. In other words, the protective effect induced by BAT transplantation was not seen when the obese mice were anticoagulated with transplantation of WAT under AO stimulation (Fig. S4). Some researchers have pointed out that high doses of AO (exceeding 40 mg/kg) exerted liver toxicity, however, the doses of AO used in our study were significantly lower than those reported in previous studies [41]. The differing outcomes of transplantation highlight BAT as a potential therapeutic target for AO in alleviating obesity. Therefore, these results collectively suggest that the effect of AO on the interaction between BAT and the liver warrants further investigation.

To confirm the substrates and transport mode released from BAT to liver, we incubated mouse hepatocytes with CM collected from lipid- and AO-treated BAT adipocytes as well as incubated with DNase, RNase, or GW4869. Interestingly, DNase but not RNase, resulted in the removal of the OAPA attachment and a decrease in the levels of inflammatory factors, which can synergistically increase the efficacy of AO (Fig. 5). More investigation have shown that the mtDNA released from overwhelmed BAT is released and distributed to other tissues and can disseminate to neighboring cells through EVs generated by the mitochondria, which subsequently triggers the induction of oxidative processes [42]. Moreover, adipose tissue transfers inflammatory EVs to the liver to support hepatic lipid balance and aberrant repair after prolonged lipid overload [43]. Furthermore, a specific EV inhibitor GW4869 resulted in the loss of EV released from BAT and significantly inhibits the production of inflammatory factors in hepatocytes (Fig. 5). Moreover, we also isolated EVs from the BAT cells with different stimulation and injected into mice by tail vain injection. Interestingly, CM supernatant after ultra-high-speed centrifugation (same method for collecting EVs) from BAT cells treated with GW4869 no longer caused significant pathological damage to the liver and showed lower hepatic expression of inflammatory factors that mice treated with OAPA-EVs, suggesting that mtDNA from BAT can be transported by EVs that aligned with the findings of our in vivo evaluation. Similar protective results were also found in Sting^−/−^ mice, suggesting that sting is a key downstream target for communication between BAT and liver. Our future studies will investigate whether AO stimulates liver-derived substances that influence BAT function and how this interaction further modulates inter-organ communication between these two organs.

## Conclusion

In conclusion, our findings demonstrated that AO inhibited the opening of the mPTP and reduced the release of mtDNA from overwhelmed BAT cells in obesity by decreasing CypD acetylation, upregulating mitophagy and suppressing oligomerization of VDAC1. Moreover, AO reduced the level of PCSK9 to regulate ANT and VDAC1 and subsequent inhibition on mPTP. Thus, AO abrogated the transmission of damage-associated DNA-enriched EVs from BAT to liver, which contributes to hepatic inflammation. Our study provides novel insights into the mechanisms underlying the anti-obesity effects of AO from the perspective of the BAT-Liver axis, further suggesting the promising prospects of AO in the field of obesity treatment.

## Supplementary Information

Supplementary material 1

## Data Availability

All data included in this article are available from the corresponding author.

## Abbreviations

ALT: Alanine aminotransferase
ANT: Adenine nucleotide translocase
AO: Aurantio-obtusin
AST: Aspartate aminotransferase
BAT: Brown adipose tissue
CypD: Cyclophilin D
cGAS: Cyclic GMP-AMP synthase
EVs: Extracellular vesicles
HFHS: High-fat diet and glucose-fructose water
mtDNA: Mitochondrial DNA
mPTP: Mitochondrial permeability transition pore
MDA: Malondialdehyde
NEFA: Nonesterified free fatty acid
OAPA: Oleic acid and palmitic acid
PCSK9: Proprotein convertase subtilisin/kexin type 9
PINK1: PTEN induced putative kinase 1
VDAC1: Voltage-dependent anion channel 1
ROS: Reactive oxygen species
SOD: Superoxide dismutase
STING: Stimulator of interferon genes
TG: Total triglyceride
WAT: White adipose tissue
