# Supplementary material for: Aurantio-obtusin improves obesity and protects hepatic inflammation by rescuing mitochondrial damage in overwhelmed brown adipose tissue
Source: Chin Med. 2025 Mar 25;20:41. doi: 10.1186/s13020-025-01097-y (PMC11934537; doi:10.1186/s13020-025-01097-y)
Supplement: Supplementary file 1 — Supplementary material 1 [file 13020_2025_1097_MOESM1_ESM.docx]

**Supplementary files**

1. Supplementary materials and methods

**1.1 Measurement of** **total triglyceride (TG), total cholesterol (TC), alanine aminotransferase (ALT), aspartate aminotransferase (AST) and nonesterified Free fatty acids (NEFA) level in serum and different tissues**

The liver, BAT and blood were obtained after mice were sacrificed. Then, the mice’s varied indexes from different samples were detected using TG assay Kit (A110-1-1), TC assay kit (A111-1-1), ALT assay kit (C009-2-1), AST assay kit (C010-2-1) and NEFA assay kit (A042-2-1) according to the manufacturer’s instructions. The above kits were obtained from Nanjing Jiancheng Bioengineering Institute (Nanjing, China).

**1.2 Measurement of** **superoxide dismutase (SOD) and malondialdehyde (MDA)**

BAT tissues were homogenized using normal saline to obtain supernatant. Cell samples were lysed in RIPA lysis buffer. Then processed samples prepared the supernatant for subsequent detection. SOD assay kit (A001-3-2) and MDA assay kit (A003-1-2) were acquired from Nanjing Jiancheng Bioengineering Institute (Nanjing, China) and used to measure the vitality of SOD and the content of MDA according to the manufacturer’s instructions.

**1.3 Western blot analysis**

The cells and tissues were lysed with RIPA lysis buffer and protein quantitation was conducted with the BCA [protein assay](https://www.sciencedirect.com/topics/biochemistry-genetics-and-molecular-biology/protein-assay) kit ([BN27109](http://www.biodee.net/Show/index/cid/100/id/22262.html), Beijing BioDee Biotech). The protein samples were subjected to different concentrations of SDS-PAGE [gel electrophoresis](https://www.sciencedirect.com/topics/biochemistry-genetics-and-molecular-biology/gel-electrophoresis) and successively incubated at 4°C overnight with the corresponding primary antibodies. Immunoblots were developed using ECL western bolting detection reagents and visualized by ChemiDocTM Touch Imaging System (Bio-Rad, Hercules, CA). The antibodies used in [Western blot](https://www.sciencedirect.com/topics/biochemistry-genetics-and-molecular-biology/western-blot) were shown in Table 1.

**1.4** **Quantitative real-time PCR (qPCR)**

Total RNA was isolated from the tested tissues or cultured cells using Trizol reagent and was then reverse transcribed into cDNA using HiScript Ⅲ 1st Strand cDNA Synthesis Kit (R312-01, azyme Biotech). QRT-PCR was performed with AceQTM Universal SYBR qPCR Master Mix (Q511-02, Vazyme Biotech). The information about used qPCR primers were: Rn18s, GGACACGGACAGGATTGACAGATTG (forward) and TAACCAGACAAATCGCTCCACCAAC (reverse); Mt-Nd3, GTTGCATTCTGACTCCCCCAA (forward) and GACGTGCAGAGCTTGTAGGG; Mt16S, TTGTACCTTTTGCATAATGAACTAACT (forward) and TTTGCCACATAGACGAGTTGA (reverse); CytoCox, ACCAAGGCCACCACACTCCT (forward) and ACGCTCAGAAGAATCCTGCAAAGAA (reverse); HPRT1, CCGAGGATTTGGAAAAAGTGTT (forward) and CATCTCCTTCATGACATCTCGA (reverse); Sting, GGTCACCGCTCCAAATATGTAG (forward) and CAGTAGTCCAAGTTCGTGCGA (reverse); IL-1β, AATCTCACAGCAGCACATC (forward) and AGCAGGTTATCATCATCATCC (reverse); IL-6, TCTGCAAGAGACTTCCATCCAG (forward) and ATAGACAGGTCTGTTGGGAGTG (reverse); TNF-α, GTCCCCAAAGGGATGAGAAGT (forward) and TTTGCTACGACGTGGGCTAC (reverse); cGAS, AGGAACCCTGAAGAAATCTCTGTGG (forward) andCCAGCCAGCCTTGAATAGGTAGTC (reverse); Pcsk9, ACATTGTGGTGCTGATGGAGGAG (forward) and CCAAGAAGCCAGGGAAGAGGTC (reverse); Sirt3, TCTATACACAGAACATCGACGG (forward) and GCATGTAGCTGTTACAAAGGTC (reverse); Slc25a4, AAAGGCATCATTGATTGTGTCG (forward) and GTACTTGTCTTTGAAGGCGAAG (reverse); Slc25a5, CTCCCAGATCCCAAGAATACTC (forward) and AACCGTGTCAAAAGGATAGGAA (reverse); Vdac1, AGTAACACTCGCTTCGGAATAG (forward) and TGGTTTTAGGGTCTGAGTGTAC (reverse); PINK1, CATCGCCTATGAAATCTTTGGG (forward) and AATTTCAGGTTCTTCAGGGCTA (reverse); PARKINN, TTTTTCATCTACTGCAAAGGCC (forward) and TTGGAATTAAGACATCGTCCCA (reverse); FUNDC1, TGGCGTGACTGGCTGGTG (forward) and GCTTGATAAAGTCTGTTGCCTGAAG (reverse); GPX-1, GTTTGAGAAGTGCGAAGTGAAT (forward) and CGGAGACCAAATGATGTACTTG (reverse); Atg7, GTGTACGATCCCTGTAACCTAG (forward) and GATGCTATGTGTCACGTCTCTA (reverse); Pten, TGAAGACCATAACCCACCACAGC (forward) and TCATTACACCAGTCCGTCCCTTTC (reverse).

1. Supplementary Table 1. Information about antibodies used in this study

| **Antibody** | **Catalog Number** | **Vendor** |
| --- | --- | --- |
| IL-6 | sc-32296 | Santa Cruz Biotechnology (Texas, USA) |
| ATG5 (C-1) | sc-133158 | Santa Cruz Biotechnology (Texas, USA) |
| ATG7 (B-9) | sc-376212 | Santa Cruz Biotechnology |
| Parkin (PRK8) | sc-32282 | Santa Cruz Biotechnology |
| VDAC1 (B-6) | sc-390996 | Santa Cruz Biotechnology (Texas, USA) |
| 8-OHdG (15A3) | sc-66036 | Santa Cruz Biotechnology |
| PCSK9 (F-8) | sc-515082 | Santa Cruz Biotechnology |
| IL-1β (11E5) | sc-52012 | Santa Cruz Biotechnology |
| CD9 (C-4) | sc-13118 | Santa Cruz Biotechnology |
| COXIV | 11242-1-AP | Proteintech Group |
| ANT1/2 | 17796-1-AP | Proteintech Group |
| PINK1 | 23274-1-AP | Proteintech Group |
| LC3 | 14600-1-AP | Proteintech Group |
| P62 | 18420-1-AP | Proteintech Group |
| STING | 19851-1-AP | Proteintech Group |
| HSP70 | 10995-1-AP | Proteintech Group |
| Calnexin | 66903-1-lg | Proteintech Group |
| CD 63 | ab216130 | ABCAM |
| Anti-Ubiquitin (linkage-specific K63) | ab179434 | ABCAM |
| PPID | 12716-1-AP | Proteintech Group |
| NF-KB p65 | 10745-1-AP | Proteintech Group |
| ATP5A1 | 66037-1-lg | Proteintech Group |
| Anti-Histone H3 (acetyl K27) | AB177178 | ABCAM |
| SIRT3 (F-10) | sc-365175 | Santa Cruz Biotechnology |
| TOM20 (F-10) | sc-17764 | Santa Cruz Biotechnology |
| Beta Actin | 66009-1-lg | Proteintech Group |
| ANTI-DNA | CBL186 | Millpore |
| Goat anti-mouse IgG (H+L) Highly Cross-Adsorbed secondary antibody (Alexa Fluor Plus 488) | Ul287767 | Thermo Fisher Scientific |
| Goat anti-rabbit IgG (H+L), F(ab')2 Fragment (Alexa Fluor 594 Conjugate) | 8889S | Cell Signaling Technology |

1. Supplementary figures and legends


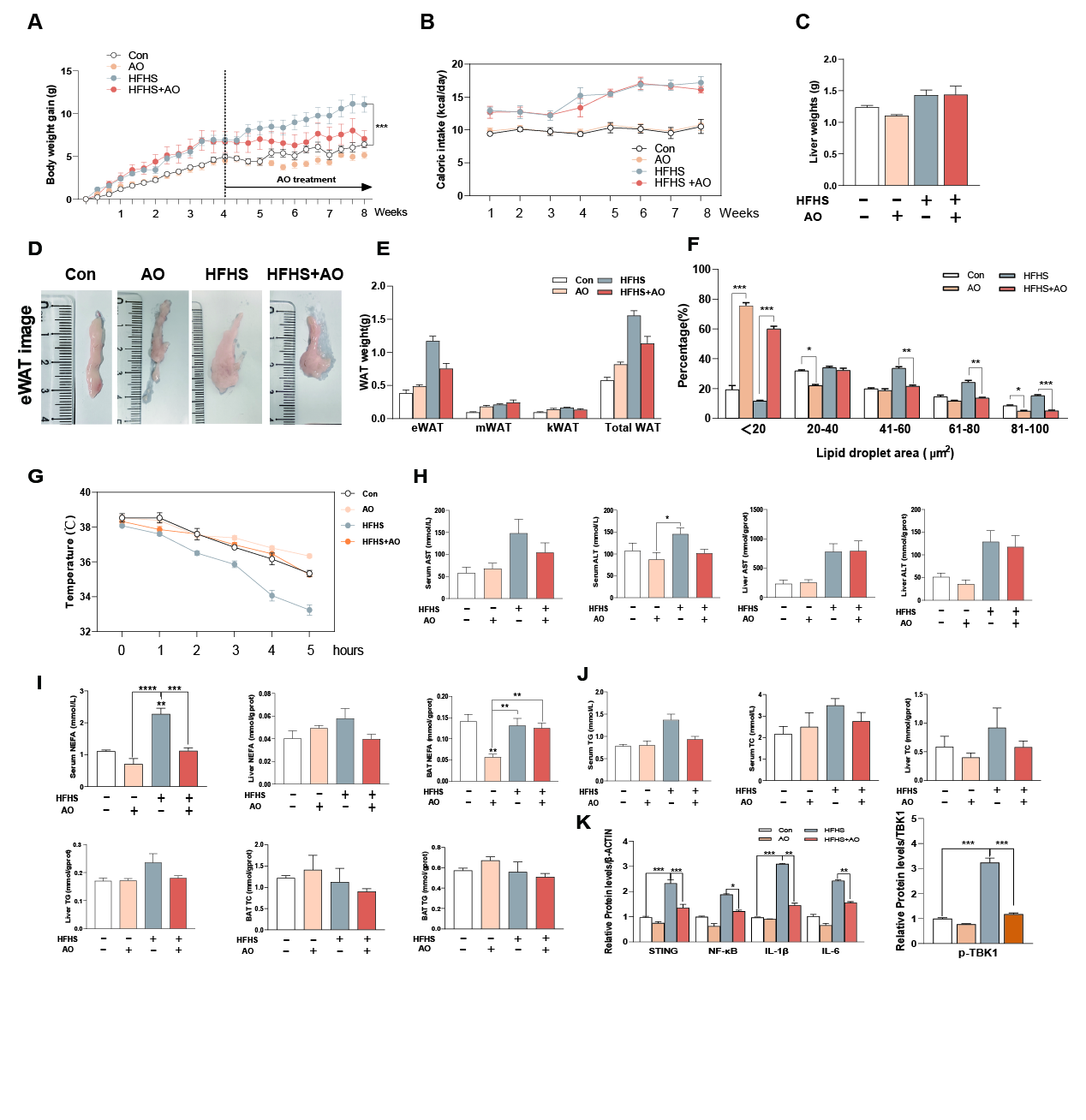


**Fig. S1. AO enhances lipid metabolism and reduces liver inflammation in mice. (A)** Body weight gain. **(B)** Caloric intake. (**C**) Liver weights of mice. (**D**) Representative images of epididymal white adipose tissue (eWAT). (**E**) Weight mass index of WAT in different mice. (**F**) The lipid droplet size in the BAT. (**G**) The rectal temperature before and after acute freezing stress test. (**H**) AST and ALT levels in serum and liver. (**I**) Serum, hepatic and BAT NEFA levels. (**J**) TC, TG levels in serum, hepatic and BAT. (**K**) Relative protein expression of STING, NF-ĸB, IL-1β and IL-6 were normalized with β-ACTIN and relative protein expression of P-TBK1 was normalized with TBK1. Statistical significance: **P* < 0.05, ***P* < 0.01, ****P* < 0.001, compare between groups (n = 6).


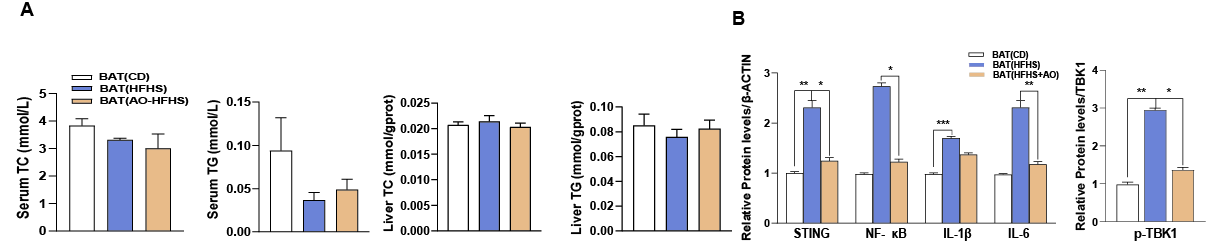


**Fig. S2. The changes of serum and hepatic TC and TG in the recipient mice after transplanting the BAT from obese or AO-treated mice.** (**A**) Serum and hepatic TC, TG levels. (**B**) Relative protein expression of STING, NF-ĸB, IL-1β and IL-6 were normalized with β-ACTIN and relative protein expression of P-TBK1 was normalized with TBK1. The abbreviations used in the figure refer to recipient mice that teceived transplants of BAT derived from control, obese, or AO-treated obese mice, namely BAT (CD), BAT (HFHS) and BAT (AO-HFHS). Statistical significance: **P* < 0.05, ***P* < 0.01, ****P* < 0.001, compare between groups (n = 6).


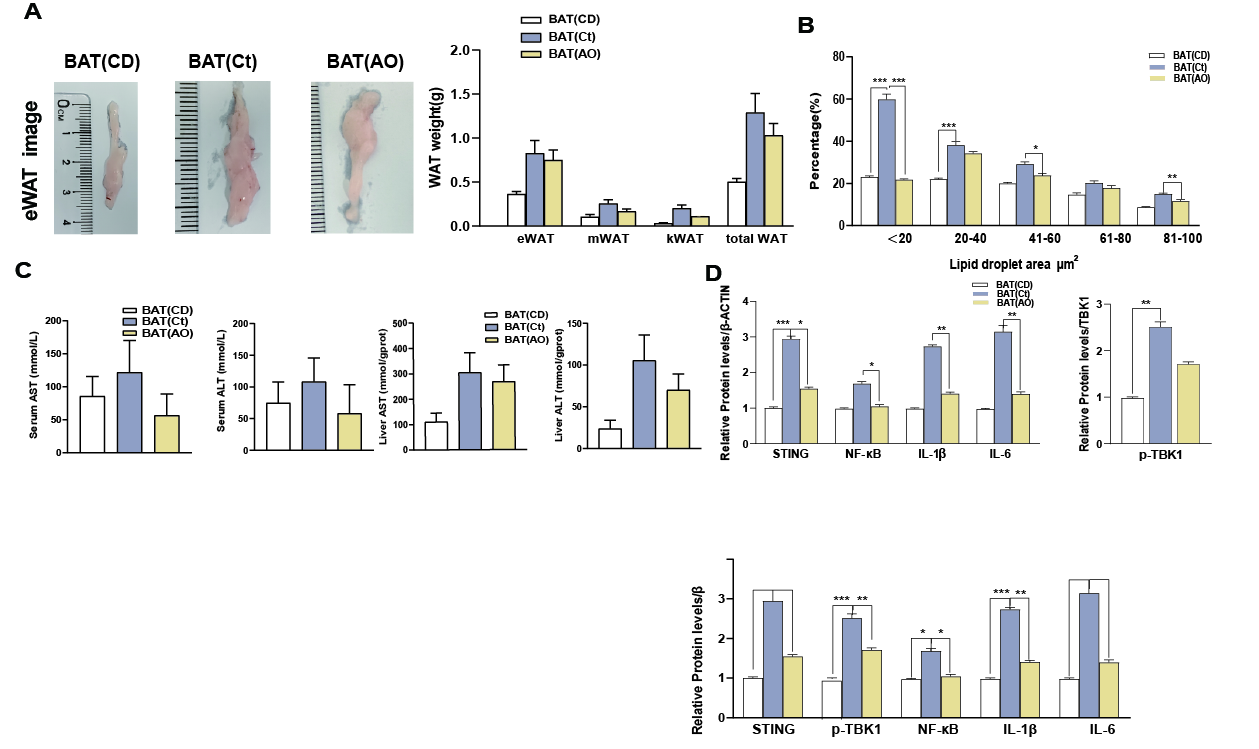


**Fig. S3. The changes of WAT and serum biochemical index in obese mice after transplanting AO-stimulated BAT.** (**A**) Representative images of epididymal white adipose tissue (eWAT) and weight mass index of WAT in mice. (**B**) The lipid droplet size in the BAT. (**C**) Serum and hepatic levels of AST and ALT (**D**) Relative protein expression of STING, NF-ĸB, IL-1β and IL-6 were normalized with β-ACTIN and relative protein expression of P-TBK1 was normalized with TBK1. The abbreviations used in the figure refer to recipient mice were fed with an HFHS diet while simultaneously transplanting BAT from control or AO-treated chow diet-fed mice, namely BAT (CD), BAT (Ct) or BAT (AO). Statistical significance: **P* < 0.05, ***P* < 0.01, ****P* < 0.001, compare between groups (n = 6).


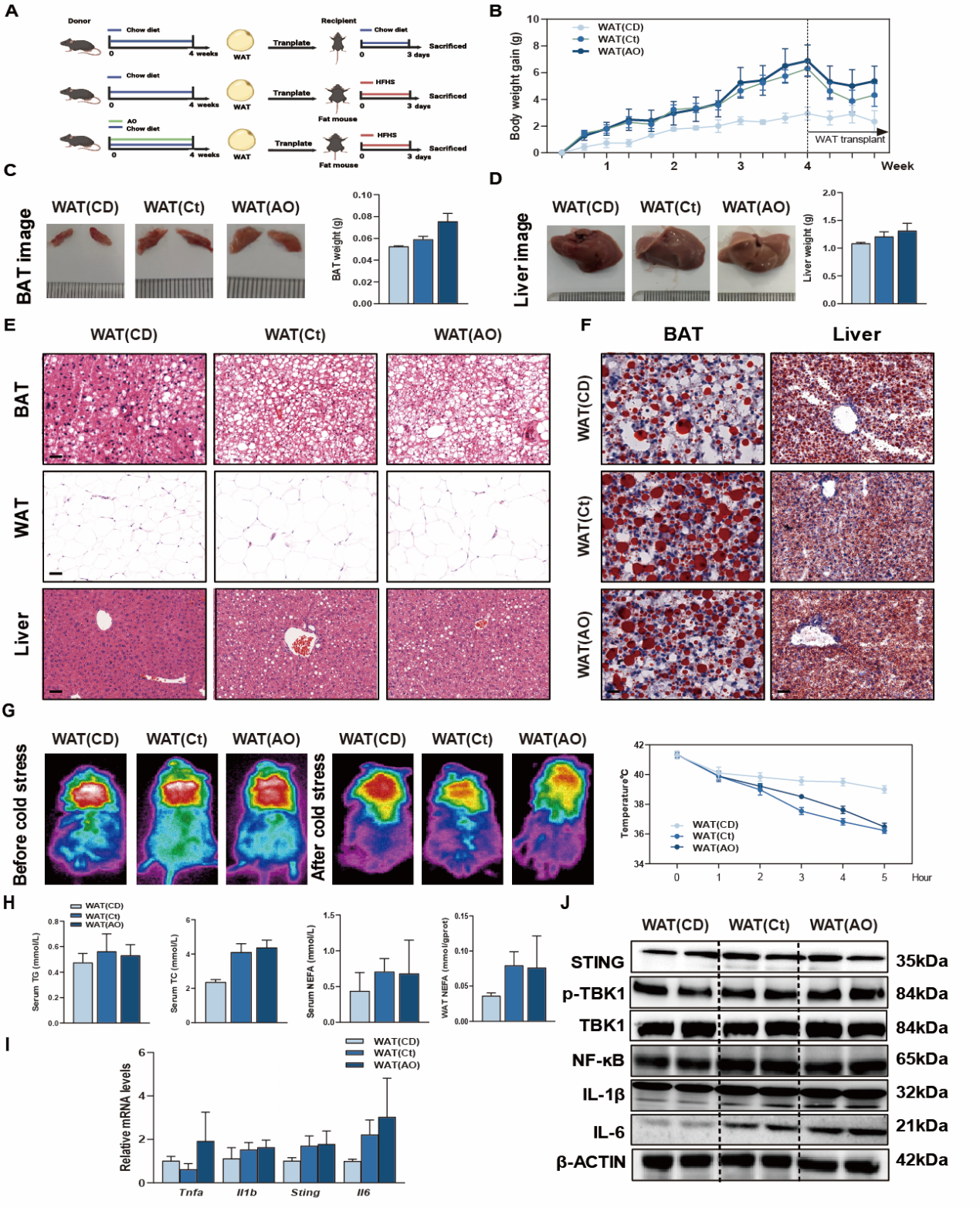


**Fig. S4. Transplantation of AO-stimulated WAT can’t alleviate liver inflammation caused by lipid toxicity.** (**A**) Mice were given with AO (10 mg/kg) for 4 weeks for WAT collection, which were then transplanted into obese mice and sacrificed after 3 days for next experimental study. (**B**) Line chart of body weight gain in mice. (**C**) Representative images of BAT and weight. (**D**) Liver images and weights. (**E**) Representative images of H & E staining of BAT, WAT and liver. Scale bar = 15 μm in BAT and WAT, scale bar = 20 μm in liver. (**F**) Oil red O staining of hepatic tissues and BAT. Scale bar = 20 μm. (**G**) Representative infrared images and the chart of rectal temperature change after freezing stress. (**H**) TG, TC, NEFA levels in WAT and serum. (**I**) The relative mRNA levels of *Sting*, *Il1b*, *Il6* and *Tnfa* in WAT were determined by qPCR and normalized using *Hprt1* as an internal control. (**J**) Representative immunoblots against STING, P-TBK1, TBK1, NF-ĸB, IL-1β, IL-6 and β-ACTIN were used as the loading control in WAT (n = 6). The abbreviations used in the figure refer to recipient mice were fed with an HFHS diet while simultaneously transplanting WAT from control or AO-treated chow diet-fed mice, namely WAT (CD), WAT (Ct) or WAT (AO).


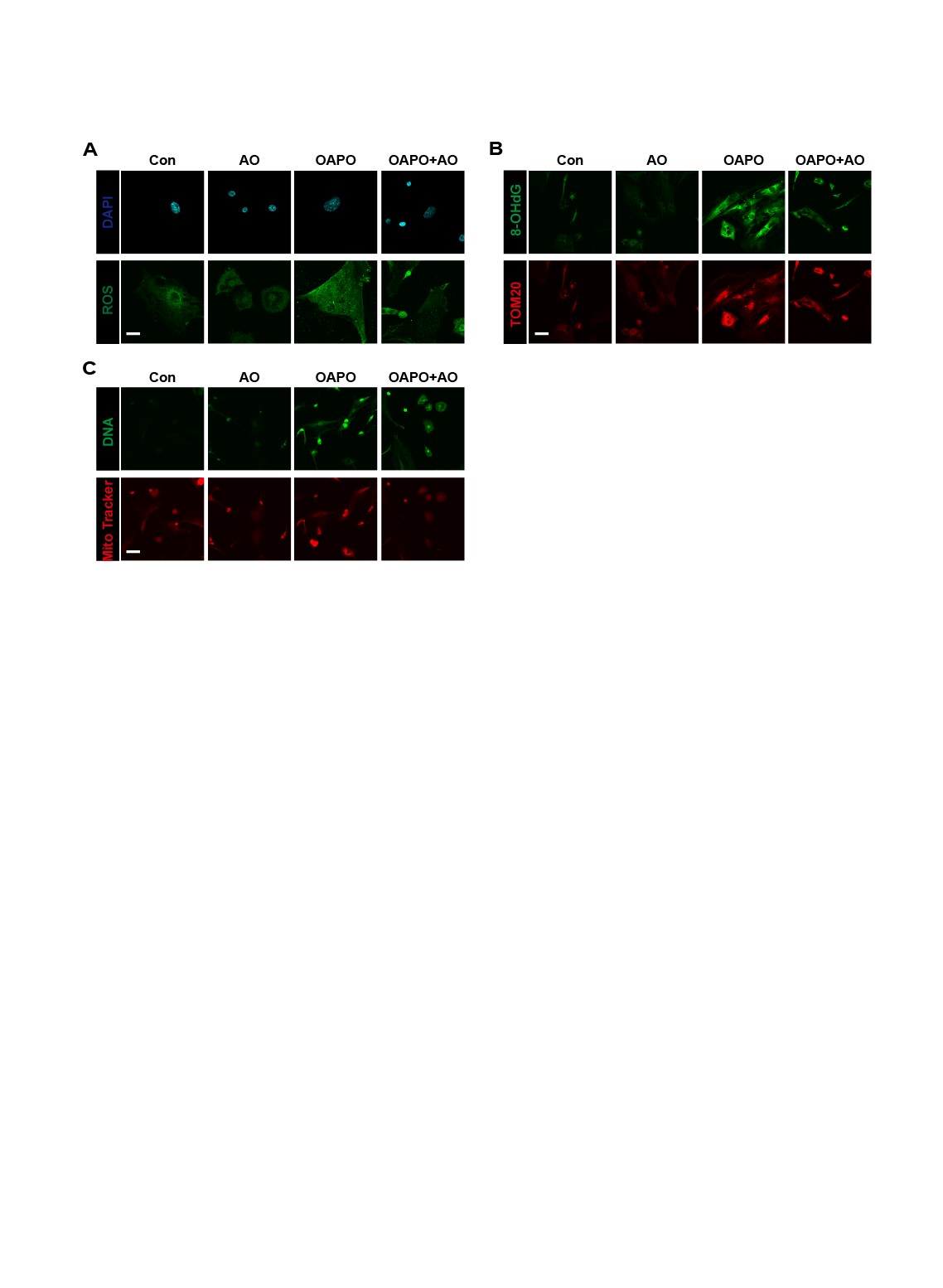


**Fig. S5.** Representative images (**A**) of immunofluorescence staining for ROS (green) in BAT primary cells. Representative images (**B**) of immunofluorescence staining for TOM20 (red) and 8-OHdG (green) in BAT primary cells. Representative images (**C**) of immunofluorescence staining for Mito Tracker (red) and DNA (green) in BAT primary cells. Scale bar = 100 µm.


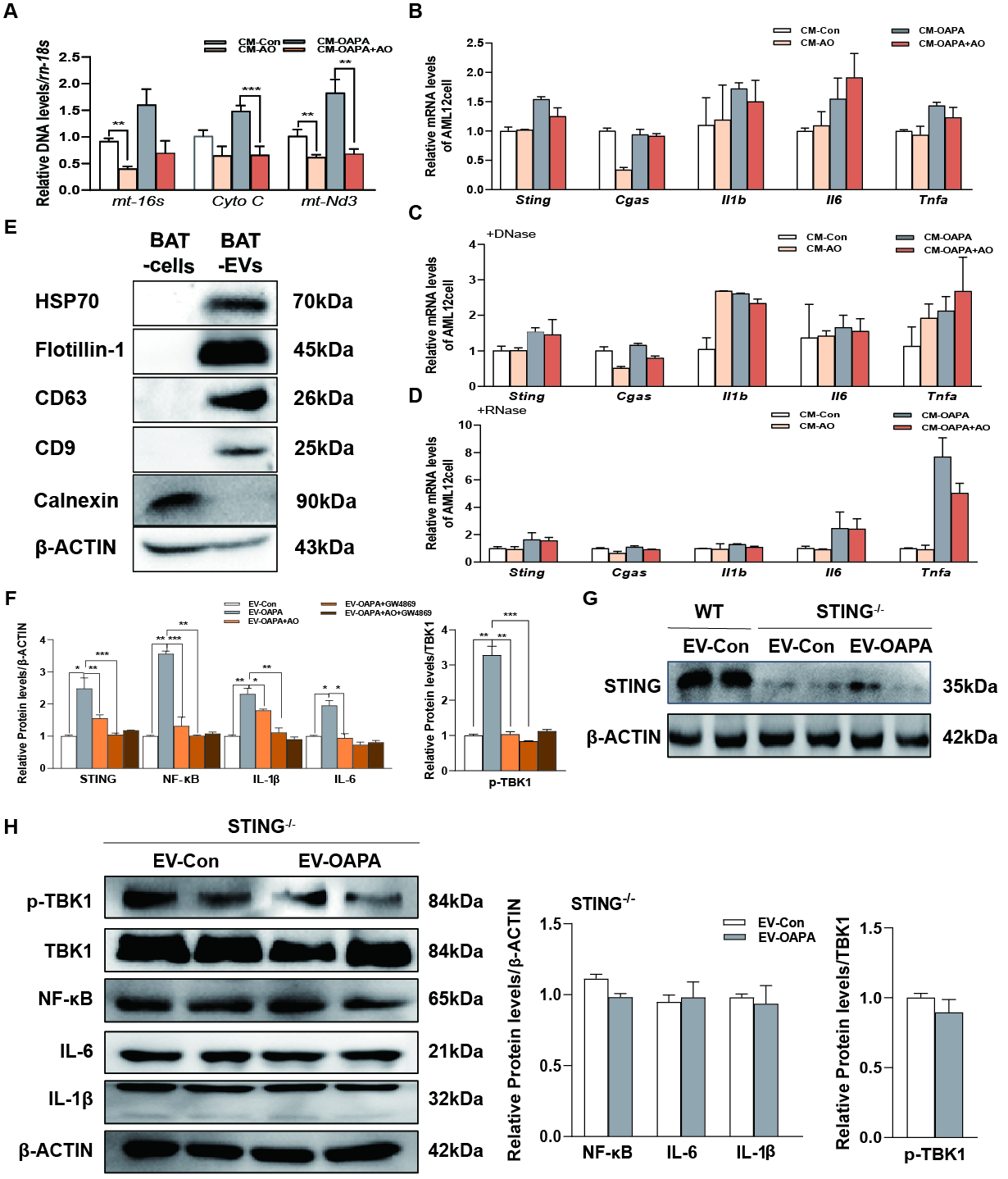


**Fig. S6.** **CM isolated from AO-treated WAT has** **little effect on the inflammatory signaling in hepatocytes*.*** (**A**) Relative mtDNA levels of *mt-16s, Cyto C* and *mt-Nd3* in CM from BAT cells. *Rn-18s* was used as an internal reference. The CM from WAT cells treated with (**B**) AO, OAPA and OAPA+AO and incubated with (**C**) DNase or (**D**) RNase were collected and given to AML12 cells for 24 h. The relative mRNA levels of *Sting*, *Cgas*, *Il1b*, *Il6* and *Tnfa* were detected by qPCR. *Hprt1* was used as an internal reference (n = 3). (**E**) Relative protein expression of HSP70, Flotillin-1, CD63, CD9 and Calnexin were normalized with β-ACTIN. (**F**) Relative protein expression of STING, NF-ĸB, IL-1β and IL-6 were normalized with β-ACTIN and relative protein expression of P-TBK1 was normalized with TBK1. (**G**) Protein levels of STING in WT and STING^-/-^ mice. (**H**) Protein levels of p-TBK1, NF-κB, IL-6, and IL-1β in STING^-/-^ mice and normalized with β-ACTIN and relative protein expression of P-TBK1 was normalized with TBK1 (n = 6). Statistical significance: **P* < 0.05, ***P* < 0.01, ****P* < 0.001, compare between groups (n = 6).


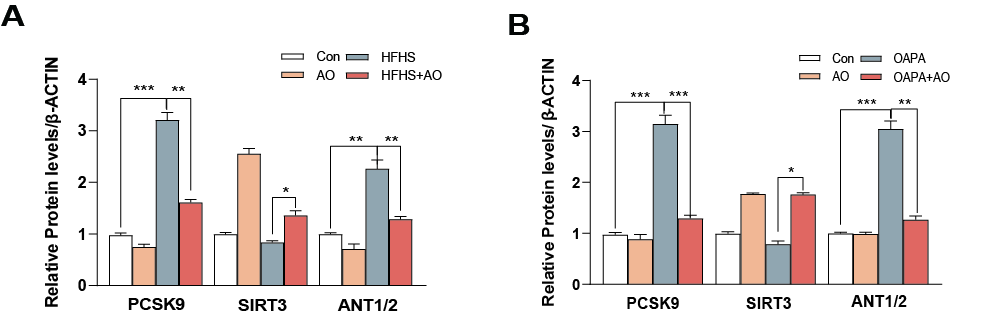


**Fig. S7.** Relative protein expression of PCSK9, SIRT3 and ANT1/2 were normalized with β-ACTIN in BAT (**A**) and BAT (**B**) cells. Statistical significance: **P* < 0.05, ***P* < 0.01, ****P* < 0.001, compare between groups (n=6 for mice and n=3 for cell experiments).

.


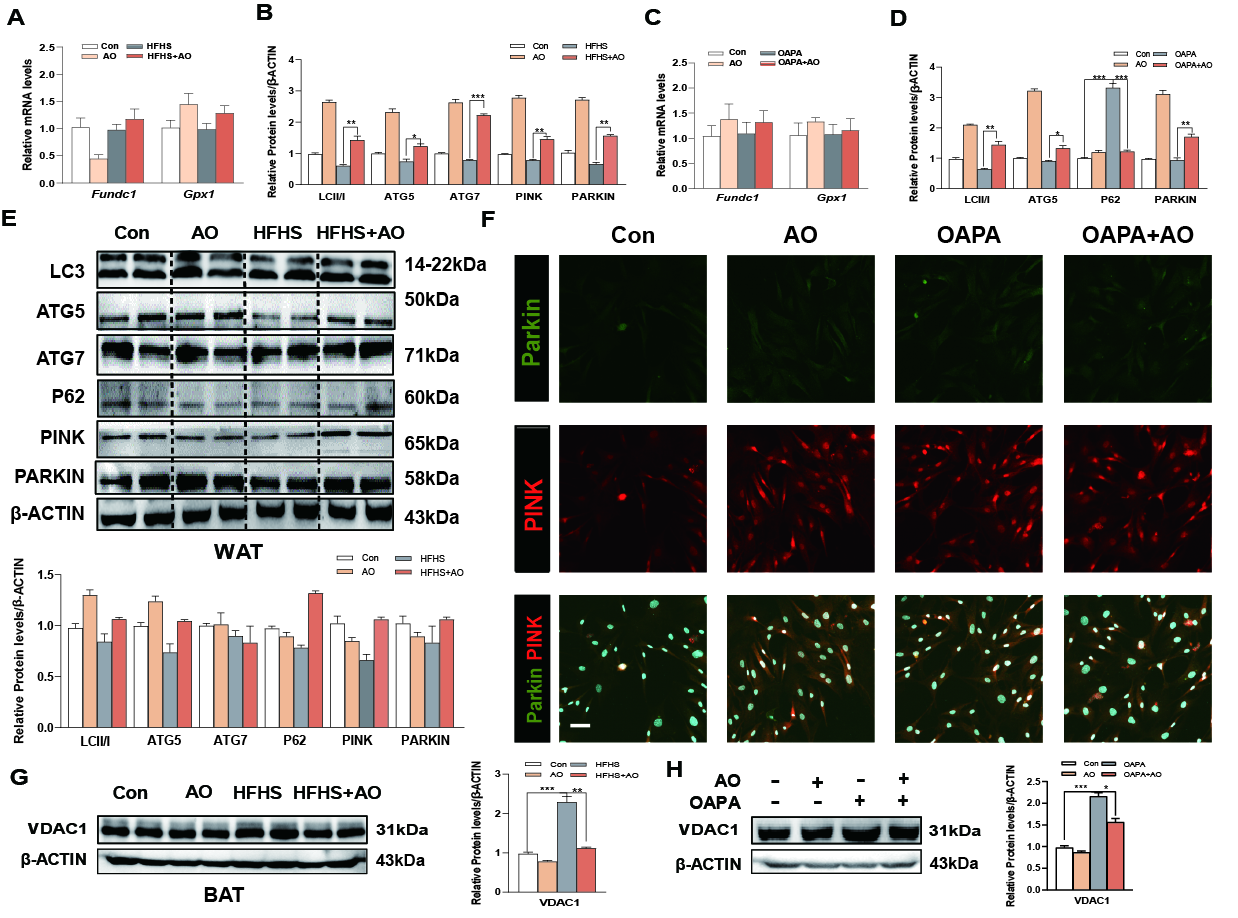


**Fig. S8. The changes of mitophagy and VDAC1 in AO-treated BAT or WAT tissues and primary cells.** The relative mRNA levels of *Fundc1* and *Gpx1* were detected by qPCR and normalized with *Hprt1* in (**A**) BAT and (**C**) BAT cells. (**B**) and (**D**) Relative protein expression of LC3Ⅱ/Ⅰ, ATG5, ATG7, PINK and PARKIN in BAT were normalized with β-ACTIN. (**F**) Representative images of immunofluorescence staining for PINK (red) and Parkin (green) in WAT primary cells. Scale bar = 100 µm. (**E**) Representative immunoblots of LC3, Atg5, Atg7, p62, PINK and Parkin in WAT tissue were detected by western blot analysis and β-ACTIN was used as the loading control. The protein levels of VDAC1 were detected by western blot analysis in (**G**) BAT tissue or (**H**) BAT cells and β-ACTIN was used as the loading control (n=6 for mice and n=3 for cell experiments).
